# Hybridization Approach to Identify Salicylanilides as Inhibitors of Tubulin Polymerization and Signal Transducers and Activators of Transcription 3 (STAT3)

**DOI:** 10.3390/ph15070835

**Published:** 2022-07-06

**Authors:** Marta Gargantilla, Leentje Persoons, Tereza Kauerová, Natalia del Río, Dirk Daelemans, Eva-María Priego, Peter Kollar, María-Jesús Pérez-Pérez

**Affiliations:** 1 Instituto de Quimica Medica (IQM, CSIC) c/Juan de la Cierva 3, 28006 Madrid, Spain; mgargantilla@iqm.csic.es (M.G.); natalia.delrio@iqm.csic.es (N.d.R.); empriego@iqm.csic.es (E.-M.P.); 2 KU Leuven Department of Microbiology, Immunology and Transplantation, Laboratory of Virology and Chemotherapy, Rega Institute for Medical Research, KU Leuven, Herestraat 49, 3000 Leuven, Belgium; leentje.persoons@kuleuven.be (L.P.); dirk.daelemans@kuleuven.be (D.D.); 3 Department of Pharmacology and Toxicology, Faculty of Pharmacy, Masaryk University, Palackeho tr. 1946/1, 612 42 Brno, Czech Republic; kauerovat@pharm.muni.cz

**Keywords:** tubulin polymerization inhibitors, colchicine site, salicylanilides, niclosamide, signal transducer and activator of transcription (STAT3) inhibitors

## Abstract

The superimposition of the X-ray complexes of cyclohexanediones (i.e., TUB015), described by our research group, and nocodazole, within the colchicine binding site of tubulin provided an almost perfect overlap of both ligands. This structural information led us to propose hybrids of TUB015 and nocodazole using a salicylanilide core structure. Interestingly, salicylanilides, such as niclosamide, are well-established signal transducers and activators of transcription (STAT3) inhibitors with anticancer properties. Thus, different compounds with this new scaffold have been synthesized with the aim to identify compounds inhibiting tubulin polymerization and/or STAT3 signaling. As a result, we have identified new salicylanilides (**6** and **16**) that showed significant antiproliferative activity against a panel of cancer cells. Both compounds were able to reduce the levels of p-STAT3^Tyr705^ without affecting the total expression of STAT3. While compound **6** inhibited tubulin polymerization and arrested the cell cycle of DU145 cells at G2/M, similar to TUB015, compound **16** showed a more potent effect on inhibiting STAT3 phosphorylation and arrested the cell cycle at G1/G0, similar to niclosamide. In both cases, no toxicity towards PBMC cells was detected. Thus, the salicylanilides described here represent a new class of antiproliferative agents affecting tubulin polymerization and/or STAT3 phosphorylation.

## 1. Introduction

**Figure 1:**
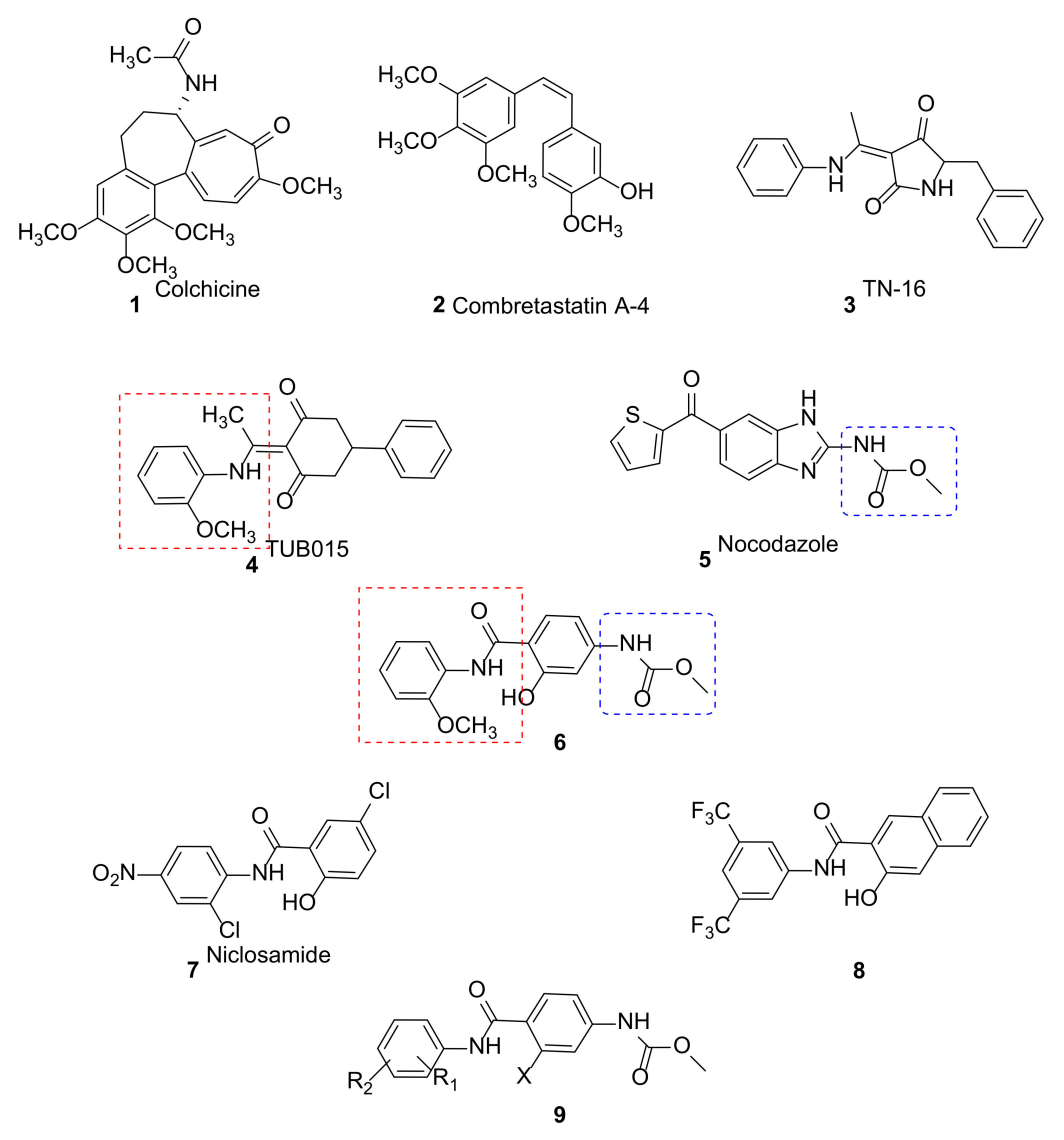
Chemical structures of colchicine-site binders in tubulin (**1**–**5**) and different salicylanilides (**6**–**9**) including niclosamide (**7**) and the here addressed compounds (**6**,**9**).

Compounds binding in the colchicine site at the αβ-tubulin interface have been extensively studied as antimitotic agents, although their therapeutic applications could be much wider than their effect on mitosis [1]. Colchicine (**1**, Figure 1) is used for the treatment of gout and familial Mediterranean fever [2]. Its anti-inflammatory properties have led to its inclusion as a therapeutic agent for COVID-19 [3], although its use outside clinical trials has been discouraged [4]. The natural stilbene combretastatin A-4 (CA-4, **2**), which also binds to tubulin at the colchicine site, has been shown to have vascular disrupting activity in the tumor microenvironment and its phosphate prodrug fosbretabulin has progressed to phase III clinical trials [5]. The therapeutic interest related to compounds binding at the colchicine site in tubulin has made these compounds an extremely active area of research, and different chemical classes have been described [6,7,8].

**Figure 2:**
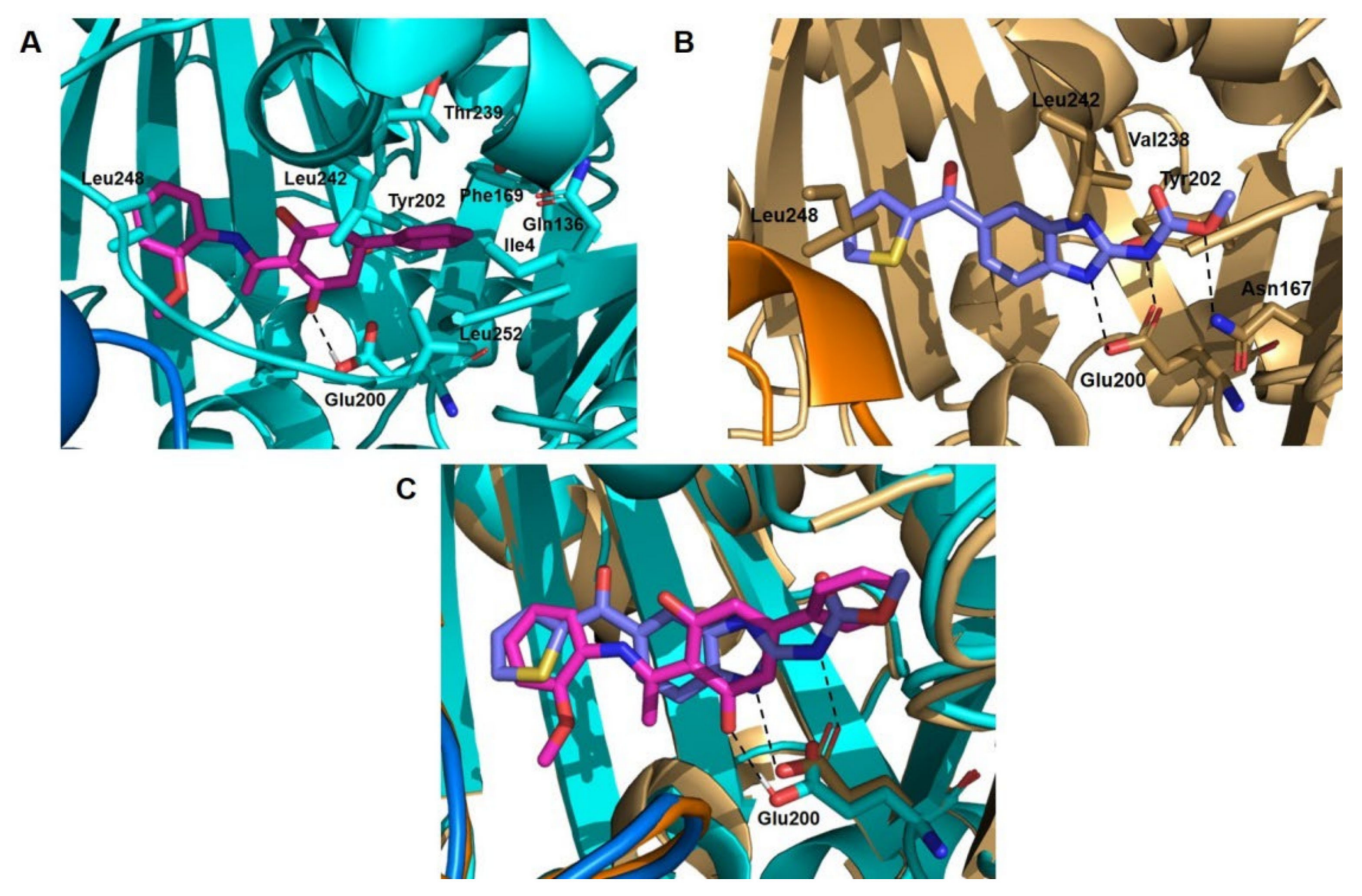
(**A**) X-ray complex structure of TUB015 (**4**, magenta sticks) in tubulin (α-tubulin shown in blue marine and β-tubulin in cyan). (**B**) X-ray complex structure of nocodazole (**5**, violet sticks) in tubulin (α-tubulin is shown in orange and β-tubulin in light orange). (**C**) Superimposition of X-ray structures of TUB015 (**4**) and nocodazole (**5**). Selected residues of the binding pocket are shown in sticks and labeled. Dashed lines represent hydrogen bonds.

Our group has been involved in the identification and optimization of new scaffolds that are able to bind at the colchicine site in tubulin. Performing a ligand-based virtual screening approach, using TN-16 (**3**) as the model compound, we identified a series of cyclohexanediones that are potent colchicine-site binders [9]. Recently, the binding mode of these cyclohexanediones [i.e., TUB015 (**4**), Figure 1] into the colchicine site in tubulin has been determined by X-ray diffraction [10]. As expected, the compound binds in a similar way to TN-16 into the deeper part of the β-subunit (Figure 2A). The 5-phenyl ring of the cyclohexanedione is lodged into a cavity lined by residues βIle4, βTyr52, βGln136, βPhe169, βTyr202, βThr239, βLeu242, and βLeu252, among others, while there is a polar interaction between one of the carbonyls of the cyclohexanedione and the side chain of βGlu200. On the other hand, the 2-methoxyaniline of TUB015 is stabilized by hydrophobic interactions. Among the published structures of colchicine ligands with tubulin, nocodazole (**5**, Figure 1) occupies the same deep pocket as TUB015, and, interestingly, it also interacts with βGlu200 (Figure 2B) [11]. Indeed, when the binding modes of TUB015 and nocodazole are superimposed, there is almost a perfect fit, as shown in Figure 2C.

In our aim to identify new scaffolds able to bind at the colchicine binding site of tubulin, we designed the hybrid molecule **6** (Figure 1), carrying a methyl carbamate as in nocodazole, an OH able to interact with βGlu200, and a 2-methoxyaniline as in TUB015. The resulting molecule **6** is a salicylanilide that can be structurally considered as a niclosamide analog. Niclosamide (**7**) is extensively studied for anticancer purposes affecting different pathways [12,13]. Among the described activities, niclosamide has been reported to inhibit the cytoplasmic signal transducer and activator of transcription-3 (STAT3) signaling [14,15], a pathway regulating important processes for tumorigenesis such as cell proliferation, cell cycle progression, or apoptosis. While in normal cells the levels of STAT3 remain transient, in a high number of solid tumors and hematological cancers, STAT3 is constitutively active [16]. Cytoplasmic STAT3 is activated through the phosphorylation at residue Tyr705 by Janus associated kinase (JAK) or other kinases, leading to its dimerization and translocation into the nucleus where STAT3 activates the transcription of a wide variety of genes related to cell proliferation and differentiation, immune response, or apoptosis among others [17]. Very recently, niclosamide has been shown to increase the efficacy of PD-1/PD-L1 immune checkpoint by the blockage of phosphorylated STAT3 to the promoter of PD-1 [18], opening new possibilities for cancer immunotherapy through targeting STAT3 [19]. 

Other salicylanilides based on hydroxynaphthalene carboxamides have also been recently described as STAT3 inhibitors [20]. Interestingly, in this naphthalene series, the best results were obtained with compound **8** (Figure 1), carrying a 3,5-diCF_3_ aniline. Thus, we hypothesized that compounds with the general structure **9**, with one or two substituents (R_1_ and R_2_) at the aniline moiety, including a 3,5-diCF_3_ substitution, may constitute a new class of antiproliferative agents by combining tubulin-binding at the colchicine site (similar to TUB015) and STAT3 inhibition based on the reported activities for niclosamide and hydroxynaphthalene derivatives exemplified by compound **8**. Additionally, the importance of the phenolic OH in this series has been studied by its replacement with F (**9**, X = F). Besides the synthesis of this new family of salicylanilides, their antiproliferative activity against a panel of cancer cell lines is reported here. Moreover, additional assays have been performed to propose tubulin and STAT3 signaling as relevant targets that account for the antiproliferative effects.

## 2. Results and Discussion

### 2.1. Synthesis

**Scheme 1:**
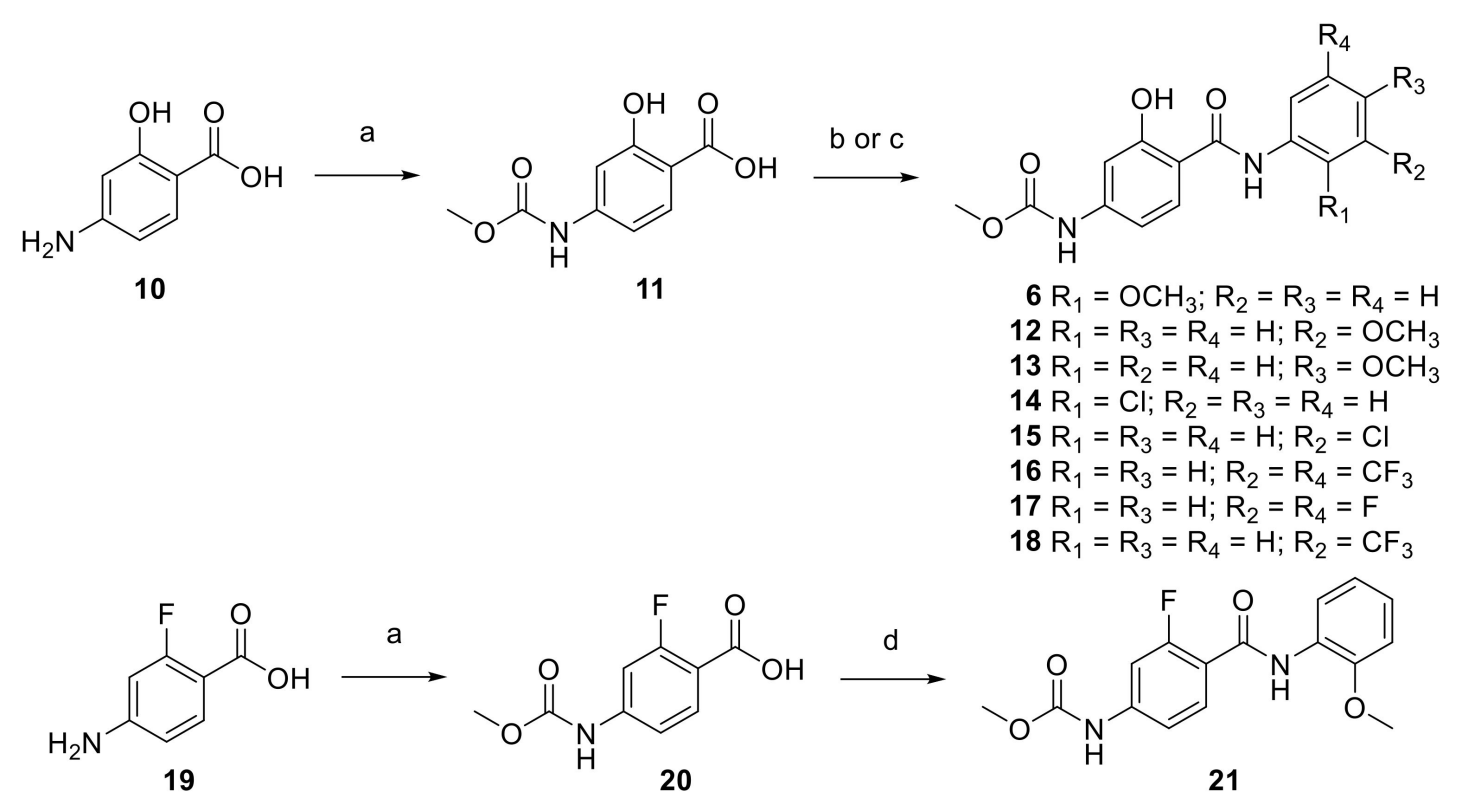
Reagents and conditions: (a) Methyl chloroformate, ethyl acetate, 80 °C, 1.5–3 h, (**11**, 95% yield; **20**, 62% yield); (b) for **6**, **12**, **13**, **15**–**17** appropriate aniline, PCl_3_, anhydrous toluene, MW: 150 °C, 10 min, (**6**, 27% yield; **12**, 43% yield; **13**, 35% yield; **15**, 34% yield; **16**, 29% yield; **17**, 35% yield); (c) for **14**, **18**: appropriate aniline, N,N′-dicyclohexylcarbodiimide, DMF, MW, 100 °C, 20 min (**14**, 35% yield; **18**, 31% yield); (d) o-anisidine, PCl_3_, anhydrous toluene, 110 °C, 3 h, 66% yield.

The synthesis of the hybrid molecule **6** was addressed by reaction of 4-amino-2-hydroxybenzoic acid (**10**) with methyl chloroformate in ethyl acetate as reported in other hydroxyanilines [21] to provide a 95% yield of carbamate **11** (Scheme 1). Further reaction of **11** with o-anisidine was performed by heating in toluene to 150 °C under MW irradiation in the presence of PCl_3_ [22] to yield the target compound **6**. Similarly, acid **11** reacted with m- or p-anisidine (PCl_3_, toluene, 150 °C, MW) to afford the salicylanilides **12** and **13** in 43 and 35% yields, respectively, where the methoxy group is located at positions 3 or 4 in the aniline moiety. In the cyclohexanedione series exemplified by TUB015, the 2-OMe could be replaced by a 2-Cl without a significant loss in antiproliferative activity [9]. Thus, a chlorine substituent was also introduced at positions 2 or 3 of the aniline in the salicylanilide series (compounds **14** and **15**). Compound **14** was obtained by reaction of **11** with 2-chloroaniline in the presence of N,N′-dicyclohexylcarbodiimide in DMF [23] after 20 min of irradiation in the MW at 100 °C, while compound **15** was obtained by the standard procedure with PCl_3_. 

Based on the antiproliferative activity and mechanism of action described for compound **8** [20], the synthesis of **16** was addressed by reaction of **11** with the 3,5-bis-(trifluoromethyl)aniline using PCl_3_. Furthermore, the 3,5-bis-fluoro derivative **17** was synthesized following the same approach. As will be later discussed, compound **16** afforded potent antiproliferative activity; thus, the monosubstituted compound with a CF_3_ at position 3 (compound **18**) was also synthesized. In all cases (**6**, **12**–**18**), the ^1^H NMR spectra registered in DMSO-d_6_ showed the presence of a broad singlet at 11.66–12.31 ppm corresponding to the phenol, as described for other salicylanilides. [24] Finally, in order to study the influence of the hydroxyl substituent of **6**, the synthesis of the corresponding fluorinated derivative was accomplished. The reaction between 2-fluoro-4-aminobenzoic acid (**19**) and methyl chloroformate provided the acid **20,** which further reacted with o-anisidine to give the benzamide **21** in 66% yield. It should be mentioned that the ^1^H NMR spectrum of compound **21** showed the signal corresponding to the NH of the amide as a doublet, with a coupling constant of 11 Hz. By recording a fluorine-decoupled ^1^H NMR spectrum, this signal was observed as a broad singlet, indicating that the splitting is due to a long-range coupling with the fluorine (see Supplementary Materials). This splitting has been reported in similar compounds [25,26].

### 2.2. Biological Evaluation

#### 2.2.1. Antiproliferative Activity

The synthesized compounds were evaluated against a panel of cancer cell lines, as shown in Table 1. Compounds TUB015, niclosamide, and nocodazole were included as reference compounds. 

Compound **6** showed marked antiproliferative activity against the different cancer cell lines tested, either carcinoma or leukemia cells, with IC_50_ values in the low µM range. Moving the methoxy substituent to position three of the aniline (**12**) resulted in an inactive compound, while the incorporation of the same substituent at position four (**13**) led to moderate antiproliferative activity. A similar trend was observed with the chloro substitution in the aniline, with the 2-Cl derivative (**14**) showing antiproliferative activity in the low µM range while the 3-Cl derivative (**15**) was inactive. Interestingly, the salicylanilide bis-substituted with a trifluoromethyl group at positions 3 and 5 (**16**) also showed clear antiproliferative activity against all the cell lines tested with IC_50_ values in the low µM range, while the 3,5-diF compound (**17**) was barely active against most cell lines tested, except for the HL-60 cell line. The monosubstitution with a CF_3_ at position 3 (**18**) led to a significantly less active compound than the 3,5-disubstituted analog **16**. Finally, the replacement of the phenolic OH in compound **6** by an F atom (**21**) resulted in the annulation of the antiproliferative activity.

Thus, compounds **6** and **16** were selected for additional assays in order to gain insights into their mechanism of action as antiproliferative agents.

#### 2.2.2. Inhibition of Tubulin Polymerization

**Figure 3:**
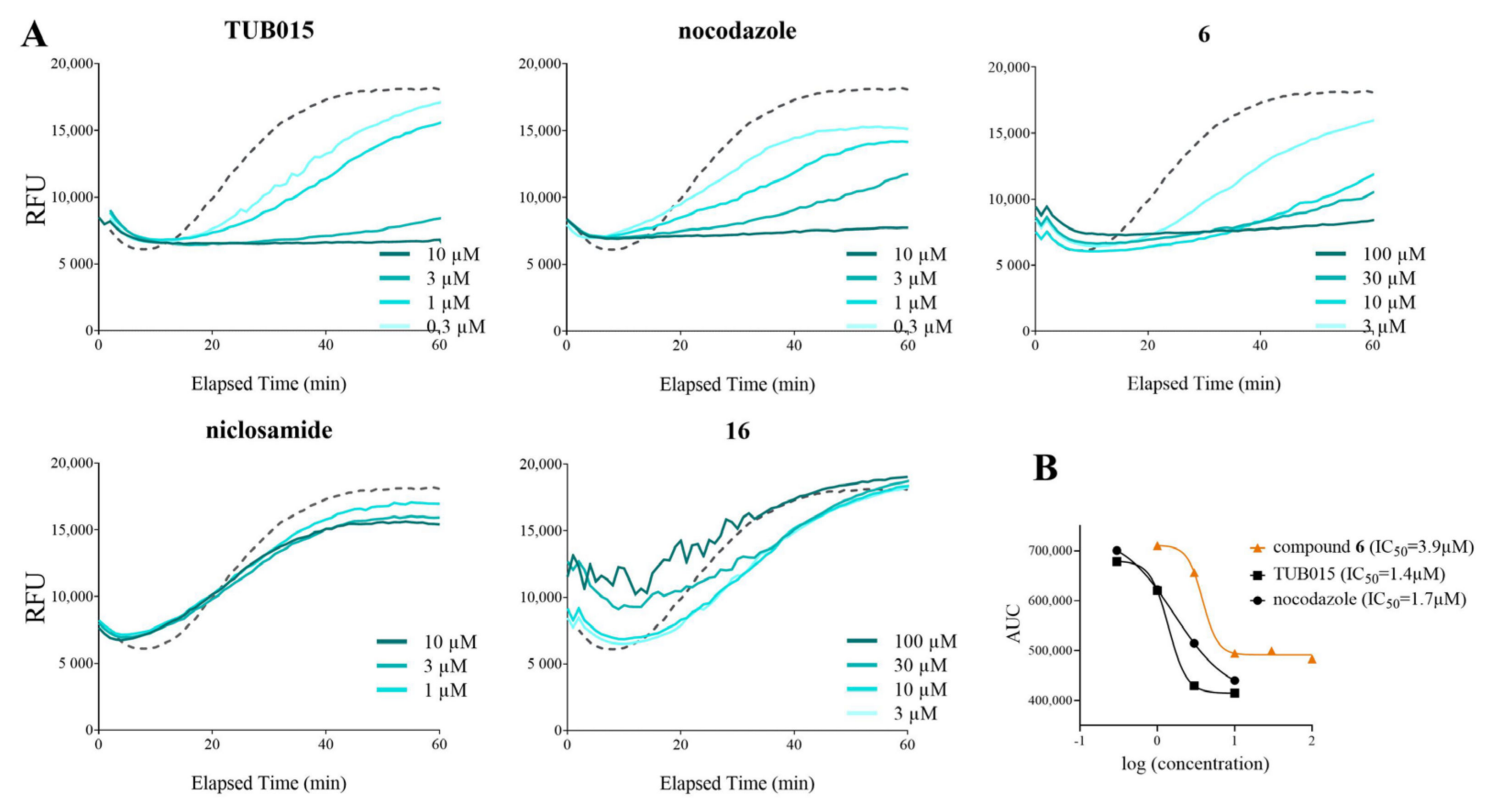
Compound **6** inhibits the rate and extent of tubulin polymerization in vitro. (**A**) Dose dependent effect on in vitro tubulin polymerization in the presence of vehicle (DMSO, dashed line) or a range of concentrations of TUB015 (**4**), nocodazole (**5**), compound **6**, (upper panel), or niclosamide (**7**) and compound **16** (lower panel). All conditions were tested in duplicate, and graphs represent average values of two (*n* = 2) independent experiments. (**B**) The measured IC_50_ values for tubulin polymerization inhibition induced by compound **6**, TUB015 (**4**), and nocodazole (**5**).

In order to determine whether the observed antiproliferative activity was due to interaction with tubulin, compounds **6** and **16**, as well as controls TUB015, niclosamide, and nocodazole were tested in a cell-free tubulin polymerization assay, as shown in Figure 3A. Polymerization of tubulin is followed by fluorescence enhancement caused by the incorporation of a fluorescent reporter into the microtubules as polymerization occurs. Without treatment (DMSO control, dashed line), tubulin subunits assemble to form microtubules in a time-dependent manner. Microtubule-destabilizing agents TUB015 and nocodazole prevent tubulin polymerization in a clear dose-dependent manner, with the highest concentration tested (10 µM) completely blocking polymerization (Figure 3A). Compound **6** exhibited a similar dose-dependent effect on tubulin polymerization confirming that compound **6** directly inhibited tubulin polymerization. The highest dose tested (100 µM) fully suppressed polymerization, and 30 and 10 µM also inhibited the polymerization almost completely. At 3 μM, compound **6** slowed down the rate of the polymerization but could not completely suppress it. The IC_50_ value of the effect of compound **6** in tubulin polymerization (3.9 µM) was comparable to those obtained for the positive control compounds TUB015 and nocodazole (Figure 3B). Compound **16** did not show a significant effect on tubulin polymerization, providing curves similar to the DMSO control or the negative control drug niclosamide.

Moreover, immunofluorescence staining of α-tubulin in HEp-2 cells illustrated that salicylanilide **6** disrupts tubulin polymerization in a dose-dependent manner, similarly to TUB015 or nocodazole (see Supplementary Figure S1), further supporting that compound **6** promotes tubulin disassembly. Docking studies performed with compound **6** using the coordinates of the TUB015-tubulin complex indicated that the compound fits in this binding site (see Supplementary Figure S2).

#### 2.2.3. Antiproliferative Activity in Human Prostate Cancer Cells DU145

As mentioned in the introduction, niclosamide and other recently reported salicylanilides (exemplified by compound **8**) have been shown to inhibit STAT3 signaling. In order to determine if our salicylanilides **6** and **16** were also interfering with this signaling pathway, proliferation assays were performed in human DU145 prostate cancer cells where STAT3 is constitutively activated [27]. As shown in Table 2, both compounds were able to considerably inhibit DU145 proliferation as determined by WST-1 analysis; this effect was most pronounced after 48 to 72 h. Compound **16** was significantly more active than compound **6** in this cell line, although both compounds showed IC_50_ values higher than those of the reference drug niclosamide.

#### 2.2.4. Effects of Compounds **6** and **16** in Regulating STAT3 Signaling Pathway In Vitro

**Figure 4:**
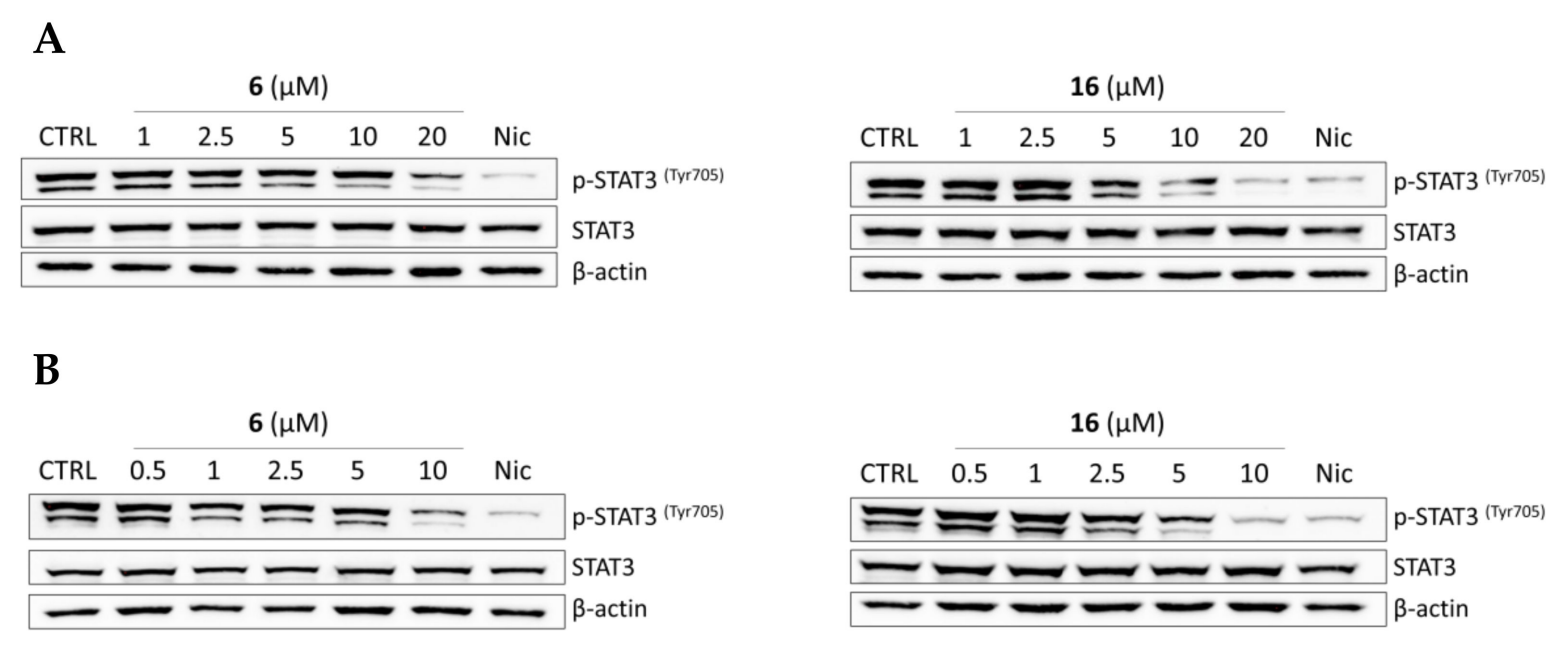
Compounds **6** and **16** reduced phosphorylation of STAT3 in human prostate cancer cells. DU145 cells were treated with both compounds at indicated concentrations for (**A**) 24 h and (**B**) 48 h. The levels of STAT3 and its phosphorylated form (p-STAT3^Tyr705^) were detected by immunoblot analysis using appropriate antibodies. Samples treated with 5 µM of niclosamide (Nic) for 24 h and 48 h were used as positive controls. Representative immunoblots of one out of three experiments are shown. CTRL, drug-free control.

To investigate the effect of compounds **6** and **16** on the regulation of STAT3 signaling, the phosphorylation of STAT3 at Tyr705 was measured in DU145 cells using niclosamide as a positive control. The levels of STAT3 and p-STAT3^Tyr705^ were examined by immuno-blot after 24 h or 48 h of treatment to better describe the kinetics of phosphorylation changes induced by the compounds under study. Both compounds were tested at concentrations ranging from 1 to 20 µM for a 24 h incubation period and from 0.5 to 10 µM for a 48 h incubation period, while niclosamide was used at a fixed concentration of 5 µM. Compounds **6** and **16** markedly reduced phosphorylation of STAT3 at Tyr705 in a concentration-dependent manner both after 24 h (Figure 4A) or after 48 h (Figure 4B), while the total expression of STAT3 was not affected.

**Figure 5:**
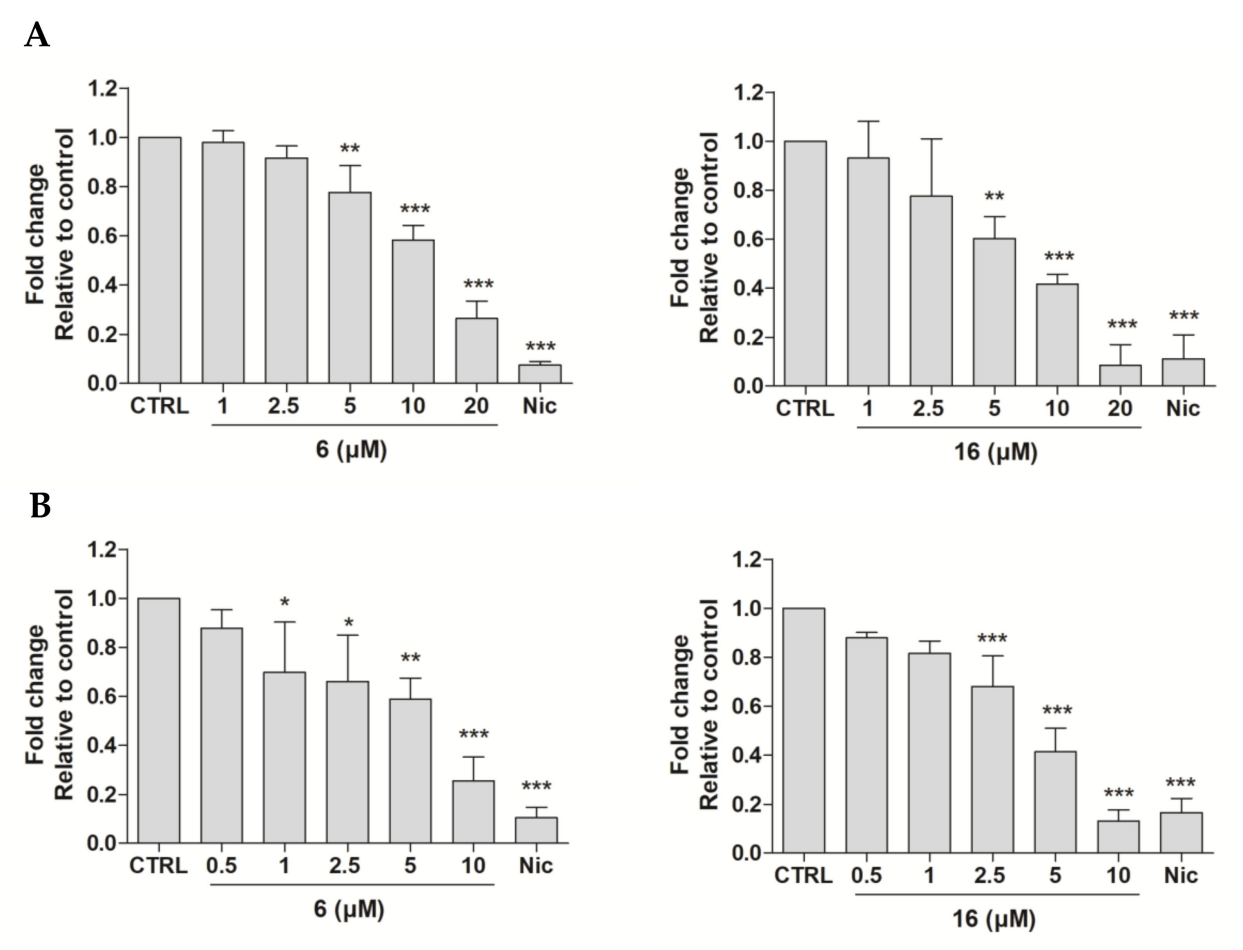
Compounds **6** and **16** reduced phosphorylation of STAT3 in human prostate cancer cells. DU145 cells were treated with both compounds at indicated concentrations for (**A**) 24 h and (**B**) 48 h. The levels of the phosphorylated form (p-STAT3^Tyr705^) were detected by immunoblot analysis using an appropriate antibody. Samples treated with 5 µM of niclosamide (Nic) for 24 h and 48 h were used as positive controls. Summary data of p-STAT3^Tyr705^ levels, where the values shown are means ± SD of three independent experiments. The levels of p-STAT3^Tyr705^ protein are expressed in relative units following normalization for the intensity of the corresponding β-actin bands. * *p* < 0.05, ** *p* < 0.01, *** *p* < 0.001, significantly different from drug-free control.

The levels of p-STAT3^Tyr705^ were quantified and represented as fold-change relative to control in Figure 5. After treatment with either compound **6** or **16**, p-STAT3^Tyr705^ phosphorylation decreased with time of exposure and concentration of the tested compounds. At both time points (24 h, Figure 5A or 48 h, Figure 5B), the effect on STAT3 phosphorylation was more significant for compound **16** than for **6**. Indeed, the reduction of the levels of p-STAT3^Tyr705^ after 48 h of treatment with compound **16** at 10 µM was very relevant, being quite comparable to the effect observed with niclosamide at 5 µM. Thus, we may conclude that both compounds are able to reduce STAT3 phosphorylation, and in this way, they might affect STAT3 signaling.

#### 2.2.5. STAT3 Translocation Experiments

**Figure 6:**
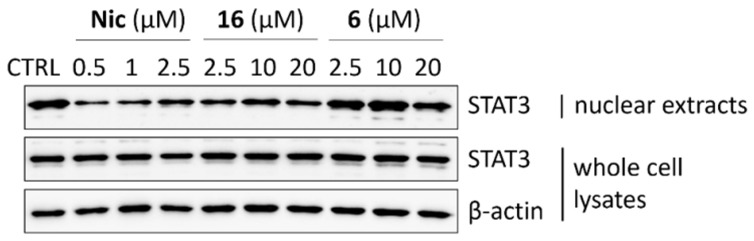
Compound **16** suppresses the EGF-induced nuclear translocation of STAT3 in human prostate cancer cells. Serum-starved DU145 cells were treated with compounds **6** and **16** at indicated concentrations for 2 h. Then the cells were stimulated with EGF (100 ng/mL) for 15 min, and the levels of STAT3 were detected by immunoblot analysis in nuclear extracts and whole cell lysates using an appropriate antibody. Samples treated by indicated concentrations of niclosamide (Nic) for 2 h were used as positive controls. Representative immunoblots of one out of at least three experiments are shown. CTRL, drug-free control.

Once phosphorylated, STAT3 forms dimers that are translocated to the cell nucleus, where it binds to specific DNA sequences and induces transcription of the target genes [28]. It has been described that niclosamide inhibits the step of phosphorylation of STAT3 and reduces STAT3 nuclear translocation [14]. Our results showed that compound **16,** similarly to niclosamide, suppressed the EGF-induced nuclear translocation of STAT3 in DU145 cells (Figure 6), while the total amount of cellular STAT3 was not affected by any of the tested compounds. Interestingly, only slight suppression of STAT3 nuclear translocation was observed upon treatment by 20 µM of compound **6**. Thus, we can assume that salicylanilide **16**, similar to niclosamide, might interfere with STAT3 signaling by suppressing its translocation into the cell nucleus.

#### 2.2.6. Effects of Compounds **6** and **16** in Cell Cycle Progression

**Figure 7:**
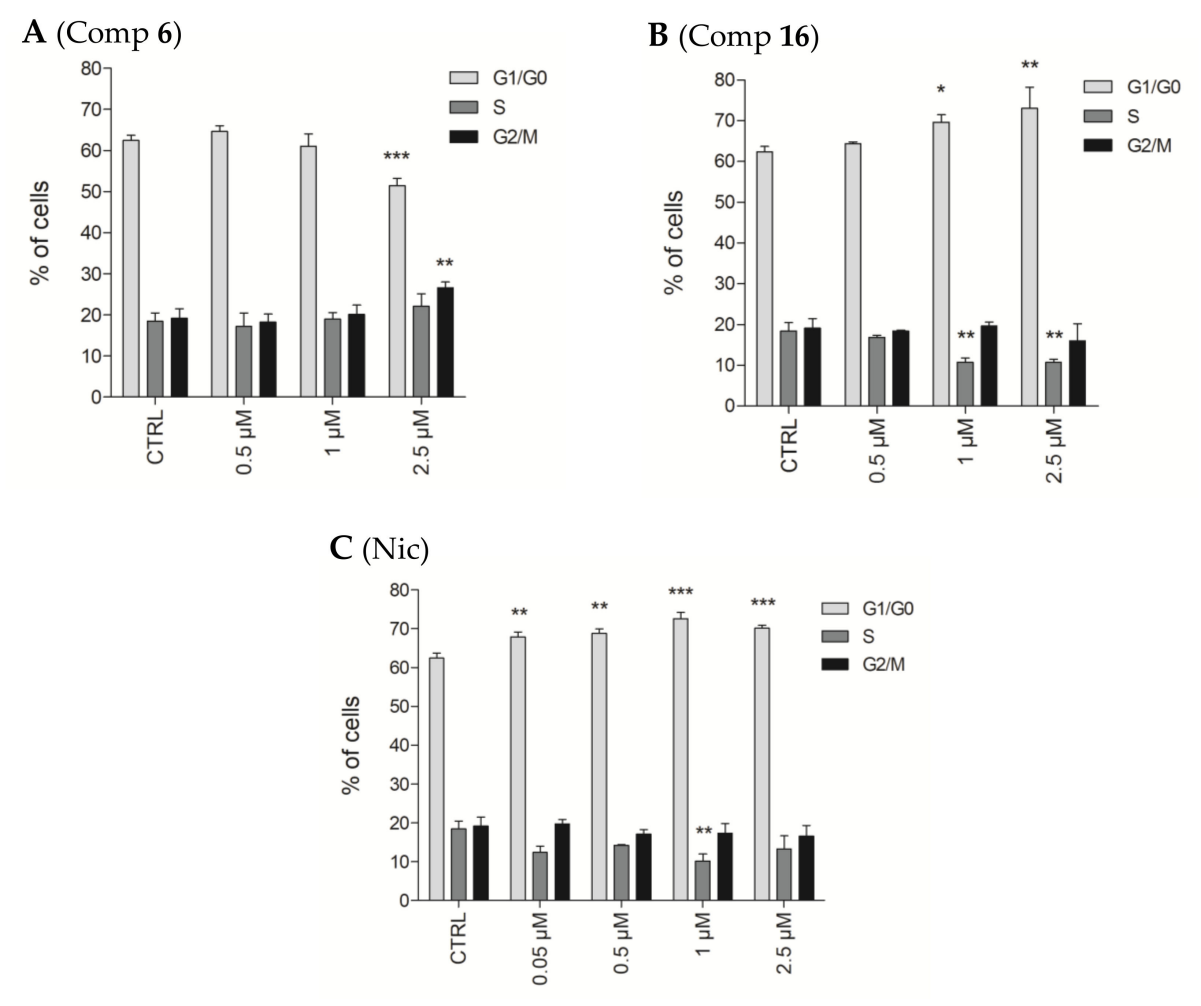
Distribution of DU145 cells in cell cycle phases after 48 h treatment with each compound. Results are expressed as the mean ± SD from three independent experiments. * *p* < 0.05, ** *p* < 0.01, *** *p* < 0.001, significantly different from drug-free control (CTRL). (**A**) shows the results obtained with **6**; (**B**), results obtained with **16,** and (**C**) results obtained with niclosamide.

Since the tested compounds showed an antiproliferative effect in DU145 cells, we further investigated how they affected progression through cell cycle phases. As seen in Figure 7A, compound **6** at 2.5 µM induced accumulation of cells in the G2/M phase as described for TUB015 and similarly to other colchicine site binders [9]. However, compound **16** increased the proportion of cells in G1/G0 phases together with a decrease in the percentage of cells in the S phase (Figure 7B). Similarly, exposure of cells to niclosamide also led to cell accumulation in the G1/G0 phase of the cell cycle (Figure 7C).

#### 2.2.7. Analysis of Apoptosis Induction in PBMC

**Figure 8:**
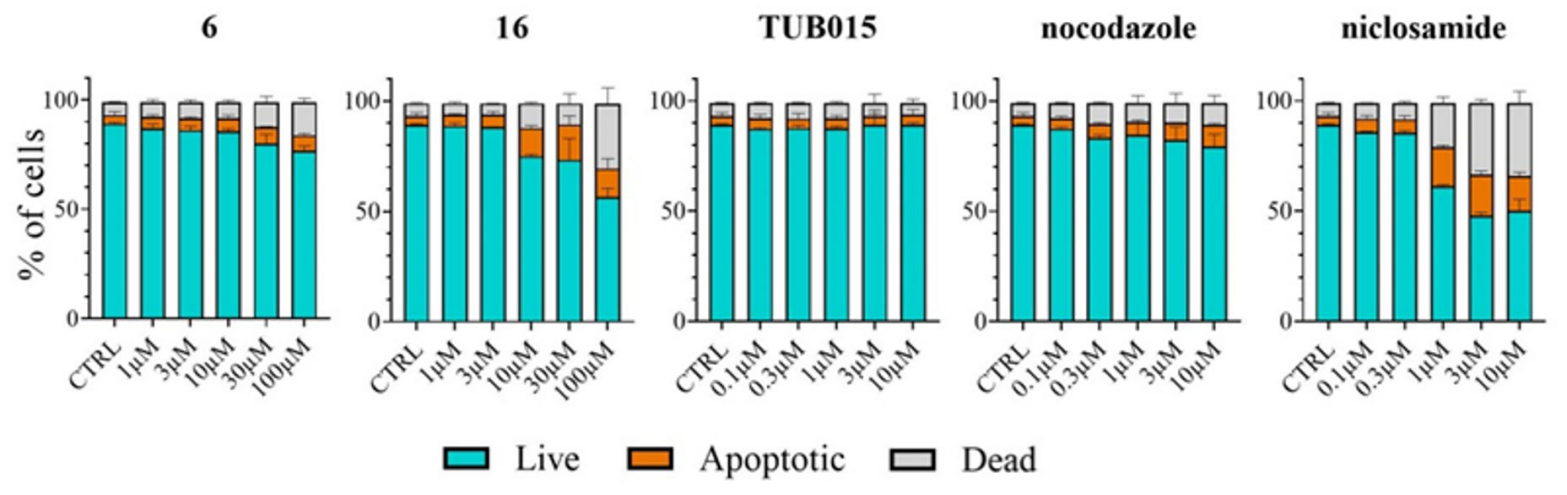
Percentages of live (cyan), apoptotic (orange), and dead (light grey) peripheral blood mononuclear cells (PBMC) after treatment for 72 h with compounds as **6**, **16**, TUB015, nocodazole, and niclosamide at the indicated concentration points. Results are reported as mean ± SD of two (*n* = 2) experiments.

Compounds **6**, **16**, TUB015, nocodazole, and niclosamide were tested in peripheral blood mononuclear cells (PBMC) in order to evaluate their selectivity toward non-cancerous cells. Compounds **6** and **16** were tested at five different concentrations ranging up to 100 µM, while for the rest of the compounds, the highest concentration tested was 10 µM. As shown in Figure 8 compound **6** did not affect the viability of normal cells, in line with the profile seen for the other microtubule targeting agents TUB015 and nocodazole. For compound **16,** an increase in the number of dead PBMCs is detected only at the highest concentration tested (100 µM). Still, this slightly toxic effect is lower than that observed with niclosamide at 10 or even 3 µM. These results indicate that compounds that mainly target microtubules showed almost no impact on the percentages of live cells even at high concentrations, while those acting as STAT3 inhibitors showed a higher proportion of dead and apoptotic cells.

## 3. Materials and Methods

### 3.1. Chemistry Procedures

Melting points were measured on an M170 apparatus (Mettler Toledo, Columbus, OH, USA) and are uncorrected. The elemental analysis was performed with a CHN-O-RAPID instrument (Heraus, Hanau, Germany). The elemental compositions of the compounds agreed within ±0.4% of the calculated values.

^1^H and ^13^C NMR spectra were recorded on a Varian INNOVA-300 (now Agilent, Santa Clara, CA, USA) operating at 299 MHz (^1^H) and 75 MHz (^13^C), respectively, and a Varian INNOVA-400 operating at 399 MHZ (^1^H) and 99 MHz (^13^C), respectively, and a VARIAN SYSTEM-500 operating at 499 MHz (^1^H) and 125 MHz (^13^C), respectively. Monodimensional ^1^H and ^13^C spectra were obtained using standard conditions.

Compounds were also analyzed by HPLC/MS with an e2695 LC (Waters, Milford, MA, USA), coupled to a Waters 2996 photodiode array detector and a Waters Micromass ZQ. The column used is a Waters SunFire C18 2.1 mm × 50 mm, 3.5 µm, and the mobile phases were A: acetonitrile and B: H_2_O, together with a constant 5% of C (H_2_O with 2% formic acid) to assure 0.1% of formic acid along the run.

Analytical TLC was performed on silica gel 60 F_254_ (Merck, Dramstand, Germany)-precoated plates (0.2 mm). Spots were detected under UV light (254 nm) and/or charring with ninhydrin or phosphomolybdic acid.

Separations on silica gel were performed by preparative centrifugal circular thin-layer chromatography (CCTLC) on a Chromatotron^R^ (Kiesegel 60 PF_254_ gipshaltig Merck), with a layer thickness of 1 and 2 mm and a flow rate of 4 or 8 mL/min, respectively.

The following is the general procedure for the reaction of 2-hydroxybenzoic acids with anilines (General procedure A): the appropriate aniline (1.0–2.0 mmol) in toluene (5–10 mL), PCl_3_ (0.5–1.0 mmol) was added to a microwaveable vial containing the corresponding 2-hydroxybenzoic acid (1.0 mmol). The reaction vessel was sealed and heated in a microwave reactor at 150 °C for 10 min. The reaction mixture was diluted with DCM (20 mL) and washed with a saturated solution of NH_4_Cl (10 mL). The organic layer was dried over Na_2_SO_4_, filtered, and evaporated to dryness. The residue was purified by CCTLC in the Chromatotron as specified.

2-Hydroxy-4-((methoxycarbonyl)amino)benzoic acid (**11**)

4-Aminosalicylic acid (**10**) (1 g, 6.53 mmol) was dissolved in ethyl acetate (45 mL). After heating to reflux for 15 min, methyl chloroformate (253 μL, 3.26 mmol) was slowly added. The final mixture was heated to reflux for 1.5 h and cooled. The mixture was filtered, and the filtrate was concentrated to yield 654 mg (95%) of compound **11** as an amorphous white solid. MS (ES, positive mode): m/z 212 (M + H)^+^. ^1^H NMR (400 MHz, DMSO-d_6_) δ: 3.69 (s, 3H, OCH_3_), 6.97 (dd, J = 8.7, 2.2 Hz, 1H, Ar), 7.15 (d, J = 2.1 Hz, 1H, Ar), 7.68 (d, J = 8.7 Hz, 1H, Ar), 10.00 (br s, 1H, NH), 11.34 (br s, 1H, OH), 13.61 (br s, 1H, COOH).

Methyl (3-hydroxy-4-((2-methoxyphenyl)carbamoyl)phenyl)carbamate (**6**)

Following the general procedure A, the acid **11** (100 mg, 0.47 mmol) and o-anisidine (64 µL, 0.57 mmol) reacted in the presence of PCl_3_ (41 μL, 0.47 mmol) in anhydrous toluene (4.7 mL). After workup, the residue was purified by CCTLC in the Chromatotron (hexane/ethyl acetate/ammonia solution 30%, 2:1:0.03) to yield 41 mg (27%) of compound **6** as a white solid. Mp 199–201 °C. MS (ES, positive mode): m/z 317 (M + H)^+^. ^1^H NMR (400 MHz, DMSO-d_6_) δ: 3.69 (s, 3H, OCH_3_), 3.87 (s, 3H, OCH_3_), 6.95 (m, 1H, Ar), 6.99 (dd, J = 8.7, 2.0 Hz, 1H, Ar), 7.06–7.08 (m, 2H, Ar), 7.34 (d, J = 2.0 Hz, 1H, Ar), 7.91 (d, J = 8.7 Hz, 1H, Ar), 8.35 (d, J = 7.7 Hz, 1H, Ar), 9.92 (br s, 1H, NH), 10.64 (br s, 1H, NH), 11.71 (br s, 1H, OH). ^13^C NMR (101 MHz, DMSO-d_6_) δ: 52.3, 56.5 (OCH_3_), 105.6, 110.1, 111.4, 113.4, 120.8, 121.0, 124.1, 128.5, 131.8, 144.1, 149.1 (Ar), 154.2 (CO), 157.6 (Ar), 163.8 (CO). Analysis calculation for (C_16_H_16_N_2_O_5_): C, 60.76; H, 5.10; N, 8.86. Found: C, 60.60; H, 5.01; N, 8.72.

Methyl (3-hydroxy-4-((3-methoxyphenyl)carbamoyl)phenyl)carbamate (**12**)

Following the general procedure A, the acid **11** (200 mg, 0.95 mmol) and m-anisidine (220 μL, 1.89 mmol) reacted in the presence of PCl_3_ (41 μL, 0.47 mmol) in anhydrous toluene (4.5 mL). After workup, the residue was purified by CCTLC in the Chromatotron (hexane/ethyl acetate/ammonia solution 30%, 1:2:0.03) to yield 132 mg (43%) of compound **12** as a white solid. Mp 196–198 °C. MS (ES, positive mode): m/z 317 (M + H)^+^. ^1^H NMR (400 MHz, DMSO-d_6_) δ: 3.69 (s, 3H, OCH_3_), 3.76 (s, 3H, OCH_3_), 6.71 (dt, J = 7.6, 2.2 Hz, 1H, Ar), 7.01 (dd, J = 8.9, 2.1 Hz, 1H, Ar), 7.19–7.29 (m, 3H, Ar), 7.37 (br s, 1H, Ar), 7.91 (d, J = 8.8 Hz, 1H, Ar), 9.94 (br s, 1H, NH), 10.20 (br s, 1H, NH), 12.08 (br s, 1H, OH). ^13^C NMR (101 MHz, DMSO-d_6_) δ: 52.3, 55.5 (OCH_3_), 105.8, 107.1, 109.4, 110.0, 111.4, 113.6, 129.9, 130.2, 139.8, 144.7 (Ar), 154.2 (CO), 159.9, 160.3 (Ar), 167.0 (CO). Analysis calculation for (C_16_H_16_N_2_O_5_·0.5H_2_O): C, 59.07; H, 5.27; N, 8.61. Found: C, 59.20; H, 5.57; N, 8.40.

Methyl (3-hydroxy-4-((4-methoxyphenyl)carbamoyl)phenyl)carbamate (**13**)

Following the general procedure A, the acid **11** (100 mg, 0.47 mmol) and p-anisidine (116 mg, 0.95 mmol) reacted in the presence of PCl_3_ (41 μL, 0.47 mmol) in anhydrous toluene (4 mL). After workup, the residue was purified by CCTLC in the Chromatotron (hexane/ethyl acetate/ammonia solution 30%, 1:1:0.02) to yield 52 mg (35%) of compound **13** as a pale yellow solid. Mp 203–205 °C. MS (ES, positive mode): m/z 317 (M + H)^+^. ^1^H NMR (400 MHz, DMSO-d_6_) δ: 3.69 (s, 3H, OCH_3_), 3.75 (s, 3H, OCH_3_), 6.94 (d, J = 9.0 Hz, 2H, Ar), 7.00 (dd, J = 8.8, 2.1 Hz, 1H, Ar), 7.18 (d, J = 2.1 Hz, 1H, Ar), 7.56 (d, J = 9.0 Hz, 2H, Ar), 7.92 (d, J = 8.8 Hz, 1H, Ar), 9.92 (br s, 1H, NH), 10.12 (br s, 1H, NH), 12.31 (br s, 1H, OH). ^13^C NMR (101 MHz, DMSO-d_6_) δ: 52.3, 55.7 (OCH_3_), 105.8, 109.3, 110.8, 114.3 123.4, 129.7, 131.4, 144.6 (Ar), 154.2 (CO), 156.4, 160.9 (Ar), 167.2 (CO). Analysis calculation for (C_16_H_16_N_2_O_5_): C, 60.76; H, 5.10; N, 8.86. Found: C, 60.69; H, 5.02; N, 8.77.

Methyl (4-((2-chlorophenyl)carbamoyl)-3-hydroxyphenyl)carbamate (**14**)

To a mixture of compound **11** (80 mg, 0.38 mmol), 2-chloroaniline hydrochloride (124 mg, 0.76 mmol) and triethylamine (106 μL, 0.76 mmol) in anhydrous DMF (1.5 mL) in a microwaveable vial, DCC (156 mg, 0.76 mmol) was added. The reaction vessel was sealed and heated in a microwave reactor at 100 °C for 20 min. The solvent was removed, and the residue was dissolved in ethyl acetate (20 mL) and washed with a saturated solution of NH_4_Cl (10 mL). The organic layer was dried over Na_2_SO_4_, filtered, and evaporated to dryness. The residue was purified by CCTLC in the Chromatotron (hexane/ethyl acetate, 3:1) to yield 42 mg (35%) of compound **14** as a white solid. Mp 197–199 °C. MS (ES, positive mode): m/z 321 (M + H)^+^. ^1^H NMR (400 MHz, DMSO-d_6_) δ: 3.69 (s, 3H, OCH_3_), 7.01 (dd, J = 8.7, 2.1 Hz, 1H, Ar), 7.15 (td, J = 7.7, 1.6 Hz, 1H, Ar), 7.34–7.40 (m, 2H, Ar), 7.54 (dd, J = 8.0, 1.5 Hz, 1H, Ar), 7.93 (d, J = 8.7 Hz, 1H, Ar), 8.38 (dd, J = 8.3, 1.6 Hz, 1H, Ar), 9.97 (br s, 1H, NH), 10.76 (br s, 1H, NH), 11.97 (br s, 1H, OH). ^13^C NMR (101 MHz, DMSO-d_6_) δ: 52.3 (OCH_3_), 105.6, 110.2, 112.6, 123.3, 123.9, 125.4, 128.3, 129.8, 131.8, 135.9, 144.6 (Ar), 154.2 (CO), 158.0 (Ar), 164.4 (CO). Analysis calculation for (C_15_H_13_ClN_2_O_4_·H_2_O): C, 53.19; H, 4.46; N, 8.27. Found: C, 53.23; H, 4.37; N, 8.24.

Methyl (4-((3-chlorophenyl)carbamoyl)-3-hydroxyphenyl)carbamate (**15**)

Following the general procedure A, the acid **11** (70 mg, 0.33 mmol) and 3-chloroaniline (70 µL, 0.66 mmol) reacted in the presence of PCl_3_ (14 μL, 0.17 mmol) in anhydrous toluene (1.7 mL). After workup, the residue was purified by CCTLC in the Chromatotron (DCM/ethyl acetate, 20:1) to yield 36 mg (34%) of **15** as a white solid. Mp 213–215 °C. MS (ES, positive mode): m/z 321 (M + H)^+^ with a Cl isotopic pattern. ^1^H NMR (400 MHz, DMSO-d_6_) δ: 3.69 (s, 3H, OCH_3_), 7.01 (dd, J = 8.8, 2.1 Hz, 1H, Ar), 7.18 (dd, J = 8.0, 2.1 Hz, 1H, Ar), 7.24 (d, J = 2.1 Hz, 1H, Ar), 7.38 (t, J = 8.1 Hz, 1H, Ar), 7.59 (dd, J = 8.1, 2.0 Hz, 1H, Ar), 7.86–7.93 (m, 2H, Ar), 9.97 (br s, 1H, NH), 10.34 (br s, 1H, NH), 11.91 (br s, 1H, OH). ^13^C NMR (101 MHz, DMSO-d_6_) δ: 52.4 (OCH_3_), 105.7, 109.5, 111.4, 119.6, 120.7, 124.1, 130.3, 130.8, 133.5, 140.2, 144.8 (Ar), 154.2 (CO), 160.1 (Ar), 167.0 (CO). Analysis calculation for (C_15_H_13_ClN_2_O_4_): C, 56.17; H, 4.09; N, 8.73. Found: C, 56.05; H, 3.95; N, 8.67.

Methyl (4-((3,5-bis(trifluoromethyl)phenyl)carbamoyl)-3-hydroxyphenyl) carbamate (**16**)

Following the general procedure A, **11** (80 mg, 0.38 mmol) and 3,5-bis (trifluoromethyl) aniline (61 µL, 0.38 mmol) reacted in the presence of PCl_3_ (31 μL, 0.38 mmol) in anhydrous toluene (3.8 mL). After workup, the residue was purified by CCTLC in the Chromatotron (hexane/ethyl acetate/ammonia solution 30%, 2:1:0.02) to yield 47 mg (29%) of compound **16** as a white solid. Mp 228–230 °C. MS (ES, positive mode): m/z 423 (M + H)^+^. ^1^H NMR (400 MHz, DMSO-d_6_) δ: 3.70 (s, 3H, OCH_3_), 7.03 (dd, J = 8.7, 2.1 Hz, 1H, Ar), 7.28 (d, J = 2.2 Hz, 1H, Ar), 7.81 (s, 1H, Ar), 7.87 (d, J = 8.7 Hz, 1H, Ar), 8.44 (s, 2H, Ar), 9.99 (br s, 1H, NH), 10.70 (br s, 1H, NH), 11.66 (br s, 1H, OH). ^13^C NMR (101 MHz, DMSO-d_6_) δ: 52.4 (OCH_3_), 105.7, 109.6, 111.5 (Ar), 117.0 (m, C4′), 187.4 (q, CF_3_), 120.9 (d, J = 4.6 Hz, C2′/C6′), 130.5 (Ar), 131.3 (q, J = 33.1 Hz, C3′/C5′), 140.9, 145.1 (Ar), 154.2 (CO), 160.0 (Ar), 167.3 (CO). Analysis calculation for (C_17_H_12_F_6_N_2_O_4_): C, 48.35; H, 2.86; N, 6.63. Found: C, 47.98; H, 3.14; N, 6.47.

Methyl (4-((3,5-difluorophenyl)carbamoyl)-3-hydroxyphenyl)carbamate (**17**)

Following the described procedure A, **11** (80 mg, 0.38 mmol) and 3,5-difluoroaniline (50 mg, 0.38 mmol) reacted in the presence of PCl_3_ (31 μL, 0.38 mmol) in anhydrous toluene (3.8 mL). After workup, the residue was purified by CCTLC in the Chromatotron (hexane/ethyl acetate/ammonia solution 30%, 2:1:0.02) to yield 42 mg (35%) of compound **17** as a white solid. Mp 231–233 °C. MS (ES, positive mode): m/z 323 (M + H)^+^. ^1^H NMR (400 MHz, DMSO-d_6_) δ: 3.69 (s, 3H, OCH_3_), 6.96 (tt, 6.96 (tt, J_H,F_ = 9.3 Hz, J_H,H_ = 2.5 Hz, 1H1H, Ar), 7.01 (dd, J = 8.7, 2.1 Hz, 1H, Ar), 7.26 (d, J = 2.1 Hz, 1H, Ar), 7.49 (dd, J_H,F_ = 9.3 Hz, J_H,H_ = 2.4 Hz 2H, Ar), 7.84 (d, J = 8.7 Hz, 1H, Ar), 9.96 (br s, 1H, NH), 10.45 (br s, 1H, NH), 11.73 (br s, 1H, OH). ^13^C NMR (101 MHz, DMSO-d_6_) δ: 52.3 (OCH_3_), 99.3 (t, J = 26.2 Hz, C4′), 103.8 (m, C2′/C6′), 105.7, 109.6, 111.8, 130.6 (Ar), 141.4 (t, J = 13.8 Hz, C1′), 144.9 (Ar), 154.2 (CO), 159.7 (Ar), 162.8 (dd, J = 242.8, 15.2 Hz, C3′/C-5′), 166.8 (CO). Analysis calculation for (C_15_H_12_N_2_O_4_): C, 55.91; H, 3.75; N, 8.69. Found: C, 55.79; H, 3.60; N, 8.58.

Methyl (3-hydroxy-4-((3-(trifluoromethyl)phenyl)carbamoyl)phenyl)carbamate (**18**)

Following a synthetic procedure similar to the one described for **14**, the acid **11** (60 mg, 0.28 mmol) and 3-(trifluoromethyl)aniline (71 μL, 0.57 mmol) reacted in the presence of DCC (117 mg, 0.57 mmol) in anhydrous DMF (1.1 mL). After workup, the residue was purified by CCTLC in the Chromatotron (DCM/ethyl acetate, 20:1). The fractions containing the compound were evaporated and the residue obtained was purified by CCTLC in the Chromatotron (hexane/ethyl acetate 1:1) to yield 31 mg (31%) of compound **18** as a white solid. Mp (decomp at 212 °C). MS (ES, positive mode): m/z 355 (M + H)^+^. ^1^H NMR (400 MHz, DMSO-d_6_) δ: 3.69 (s, 3H, OCH_3_), 7.02 (dd, J = 8.8, 2.1 Hz, 1H, Ar), 7.25 (d, J = 2.1 Hz, 1H, Ar), 7.47 (d, J = 7.8 Hz, 1H, Ar), 7.60 (t, J = 8.0 Hz, 1H, Ar), 7.87–7.96 (m, 2H, Ar), 8.19 (t, J = 2.0 Hz, 1H, Ar), 9.99 (br s, 1H, NH), 10.47 (br s, 1H, NH), 11.89 (br s, 1H, OH). ^13^C NMR (101 MHz, DMSO-d_6_) δ: 52.4 (OCH_3_), 105.8, 109.6, 111.5, 117.4 (q, J = 4.1 Hz, Ar), 120.8 (d, J = 4.0 Hz, Ar), 124.7 (q, J = 272.4 Hz, CF_3_), 124.9 (Ar), 129.8 (q, J = 31.8 Hz, Ar), 130.5, 139.7, 145.0 (Ar), 154.3 (CO), 160.3 (Ar), 167.3 (CO). Analysis calculation for (C_16_H_13_F_3_N_2_O_4_): C, 54.24; H, 3.70; N, 7.91. Found: C, 54.23; H, 3.62; N, 7.99.

2-Fluoro-4-((methoxycarbonyl)amino)benzoic acid (**20**)

As described for the synthesis of compound **11**, a mixture of 4-amino-2-fluorobenzoic acid (**19**) (500 mg, 3.2 mmol) and methyl chloroformate (126 µL, 1.6 mmol) in ethyl acetate (23 mL) was refluxed for 3 h. The reaction was filtered, and the filtrate was concentrated to yield 422 mg (62%) of compound **20** as an amorphous white solid. MS (ES, negative mode): m/z 212 (M−H)^−^. ^1^H NMR (400 MHz, DMSO-d_6_) δ: 3.71 (s, 3H, OCH_3_), 7.29 (dd, J = 8.7, 2.1 Hz, 1H, Ar), 7.45 (dd, J = 13.7, 2.1 Hz, 1H, Ar), 7.82 (t, J = 8.6 Hz, 1H, Ar), 10.23 (br s, 1H, NH), 12.90 (br s, 1H, OH).

Methyl (3-fluoro-4-((2-methoxyphenyl)carbamoyl)phenyl)carbamate (**21**)

Adapting the general procedure A, to a mixture of compound **20** (100 mg, 0.47 mmol) and o-anisidine (66 µL, 0.56 mmol) in anhydrous toluene (2.5 mL) in a pressure flask, PCl_3_ was added (83 µL, 0.94 mmol). The reaction was stirred and heated to 110 °C for 3 h. The reaction mixture was dissolved in DCM (20 mL) and washed with HCl 1M (10 mL) and NaCl (10 mL). The organic layer was dried over Na_2_SO_4_, filtered and evaporated to dryness. The residue was purified by column chromatography on silica gel (DCM/MeOH, 20:1) to yield 99 mg (66% yield) of compound **21** as a white solid. Mp 216–218 °C. MS (ES, positive mode): m/z 319 (M + H)^+^. ^1^H NMR (400 MHz, DMSO-d_6_) δ: 3.71 (s, 3H, OCH_3_), 3.87 (s, 3H, OCH_3_), 6.96 (ddd, J = 7.9, 6.6, 2.2 Hz, 1H, Ar), 7.06–7.16 (m, 2H, Ar), 7.34 (dd, J = 8.7, 2.1 Hz, 1H, Ar), 7.53 (dd, J = 14.7, 2.0 Hz, 1H, Ar), 7.86 (t, J = 8.8 Hz, 1H, Ar), 8.20 (d, J = 8.0 Hz, 1H, Ar), 9.24 (d, J = 11.3 Hz, NH), 10.23 (br s, 1H, NH). ^13^C NMR (101 MHz, DMSO-d_6_) δ: 52.6, 56.5 (OCH_3_), 105.2 (d, J = 29.9 Hz), 111.6, 114.5 (d, J = 2.5 Hz), 115.8 (d, J = 12.5 Hz), 121.0, 121.2, 125.0, 127.7, 132.3 (d, J = 3.7 Hz), 144.6 (d, J = 12.3 Hz), 149.4 (Ar), 154.2 (CO), 160.6 (d, J = 244.8 Hz, Ar), 161.5 (CO). Analysis calculation for (C_16_H_15_FN_2_O_4_): C, 60.37; H, 4.75; N, 8.80. Found: C, 60.21; H, 4.82; N, 8.64.

### 3.2. Biological Assays

#### 3.2.1. Cell Culture and Reference Compounds

Cancer cell lines Capan-1, HCT-116, NCI-H460, HL-60, K-562, and Z-138 were acquired from the American Type Culture Collection (ATCC, Manassas, VA, USA). The DND-41 cell line was purchased from the Deutsche Sammlung von Mikroorganismen und Zellkulturen (DSMZ Leibniz-Institut, Brunswick, Germany). All cell lines were cultured as recommended by the suppliers. Culture media were purchased from Gibco Life Technologies and supplemented with 10% fetal bovine serum (HyClone, GE Healthcare Life Sciences, Chicago, IL, USA).

A human prostate cancer cell line DU145 was obtained from ATCC and cultivated in RPMI 1640 supplemented with the antibiotic solution (100 U/mL of penicillin, 100 µg/mL of streptomycin (HyClone, GE Healthcare Life Sciences, Chicago, IL, USA), and 10% fetal bovine serum. Cells were maintained in a humidified incubator with 5% CO_2_ at 37 °C and were regularly tested for the presence of mycoplasma contamination.

TUB015 was obtained as previously described [9]. Niclosamide was purchased from Acros (Acros Organics, Geel, Belgium) and Abcam (Cambridge, UK), and nocodazole from TCI (TCI Chemicals, Zwijndrecht, Belgium).

Tested compounds were dissolved in dimethyl sulfoxide (DMSO) from Sigma Aldrich (St. Louis, MO, USA). The final concentration of DMSO in the assays never exceeded 0.1% (*v/v*).

#### 3.2.2. Cell Proliferation Assays

Adherent cell lines HCT-116, NCI-H460, and Capan-1 cells were seeded at a density between 500 and 1500 cells per well in 384-well tissue culture plates (Greiner). After overnight incubation, cells were treated with different concentrations of the test compounds. Suspension cell lines HL-60, K-562, Z-138, and DND-41 were seeded at densities ranging from 2500 to 5500 cells per well in 384-well culture plates containing the test compounds at the same concentration points. The plates were incubated and monitored at 37 °C for 72 h in an IncuCyte (Essen BioScience Inc., Sartorius; Göttingen, Germany for real-time imaging of cell proliferation. Brightfield images were taken every 3 h, with one field imaged per well under 10× magnification. Cell growth was then quantified based on the percent cellular confluence, as analyzed by the IncuCyte image analysis software and used to calculate IC_50_ values. Compounds were tested in at least two independent experiments and represented as mean ± SEM.

#### 3.2.3. WST-1 Analysis of Cell Proliferation

DU145 cells were seeded at 7 × 10^3^ cells/100 μL per well in 96-well plates and allowed to attach to the wells for 24 h. Cells were then treated with various concentrations of compound **6** or **16** to reach the final concentration range of 0.1–30 µM, and with various concentrations of niclosamide to reach the final concentration range of 0.01–10 µM. Cells were then incubated for 24 h, 48 h, or 72 h. Cell proliferation of DU145 cells was determined using Cell Proliferation Reagent WST-1 (2-(4-iodophenyl)-3-(4-nitrophenyl)-5-(2,4-disulfophenyl)-2H-tetrazolium) (Roche Diagnostics, Mannheim, Germany), as it was previously described [29,30]. WST-1 analysis was performed in three independent experiments, with each condition tested in triplicate. The IC_50_ values were determined using the nonlinear regression four-parameter logistic model using GraphPad Prism 5.03 software (GraphPad Software, San Diego, CA, USA).

#### 3.2.4. In Vitro Tubulin Polymerization Assay

In vitro tubulin polymerization was carried out using the fluorescence-based tubulin polymerization assay (BK011P, Cytoskeleton, Denver, CO, USA) as described by the manufacturer. Briefly, half area 96-well plates were warmed to 37 °C 10 min prior to assay start. Test compounds and reference drugs were prepared at 10× stock solutions and added in 5 µL in duplicate wells. Ice-cold tubulin polymerization buffer (2 mg mL^−1^ tubulin in 80 nM Pipes, 2 mM MgCl_2_, 0.5 mM EGTA, pH 6.9, and 10 µM fluorescent reporter + 15% glycerol + 1 mM GTP) was added into each well, followed by reading with a Tecan Spark fluorimeter in kinetic mode, 61 cycles of 1 reading per minute at 37 °C, 4 reads per well (Ex. 350 nm and Em. 435 nm).

#### 3.2.5. Tubulin Immunofluorescence Staining

HEp-2 cells (human cervix carcinoma) were seeded at 15,000 c/well in 8-well chamber slides (Ibidi). After overnight incubation, cells were treated with test compounds or reference drugs for 3 h and then fixed with 4% PFA, washed, and permeabilized. Standard immunofluorescence procedures were performed using primary mouse anti-alpha tubulin antibody (sc-5286, Santa Cruz Biotechnology) and secondary goat anti-mouse IgG conjugated to Alexa Fluor^®^ 488 (A11001, Invitrogen). Nuclei were counterstained with DAPI. Images were taken with a Leica TCS SP5 confocal microscope using an HCX PL APO 63x (NA 1.2)/water immersion objective.

#### 3.2.6. Western Blotting

Cell lysates were prepared using radioimmunoprecipitation assay (RIPA) buffer supplemented with phenylmethanesulfonylfluoride (PMSF, 1 mM), both from Cell Signaling Technology (Danvers, MA, USA) and protease and phosphatase inhibitor cocktails (Roche Diagnostics, Mannheim, Germany). SDS-PAGE and western blotting were performed according to the previously described protocols [20]. Membranes were stained with anti-β-actin antibody (Santa Cruz Biotechnology, Santa Cruz, CA, USA) to assess equal sample loading. Antibodies against STAT3 (79D7, 4904) and p-STAT3 [Tyr 705] (D3A7, 9145) were purchased from Cell Signaling Technology. The intensity of bands was semi-quantitatively analyzed using the ImageJ software (National Institute of Mental Health, Bethesda, MD, USA).

#### 3.2.7. Analysis of STAT3 Nuclear Translocation

DU145 cells were serum-starved overnight and treated with the indicated concentrations of compounds **6**, **16,** and niclosamide for 2 h. After that, the cells were stimulated with 100 ng/mL EGF (Abcam, Cambridge, UK) for 15 min. Nuclear extracts were prepared using Nuclear Extraction Kit (Abcam, Cambridge, UK) according to the manufacturer’s instructions. Whole cell lysates were prepared by use of RIPA buffer supplemented with PMSF (1 mM) and protease and phosphatase inhibitor cocktails. DU145 cells were detached with trypsin/EDTA and counted with a hemocytometer. For nuclear extract preparation, cells were centrifuged for 5 min at 1000 rpm, and the supernatant was discarded. The cell pellet was resuspended in 100 µL of 1X Pre-Extraction Buffer per 10^6^ cells and incubated on ice for 10 min. Then, samples were centrifuged for 1 min at 12,000 rpm, and the cytoplasmic extract was removed from the nuclear pellet. Extraction Buffer containing DTT and PIC was added to the nuclear pellet (10 μL per 10^6^ cells). After 15 min of incubation on ice, samples were centrifuged for 10 min at 14,000 rpm at 4 °C. Then, supernatants were collected, and the protein concentration of the nuclear extracts was measured using Roti^®^-Quant universal (Carl Roth, Karlsruhe, Germany) according to the manufacturer’s instructions. Western blotting was performed according to the previously described protocol [20].

#### 3.2.8. Cell Cycle Analysis

Sub-confluent DU145 cells were treated and subsequently incubated with indicated concentrations of compounds **6**, **16,** and niclosamide for 48 h. Cell cycle analysis was performed as it was previously described [29]. The cell cycle distribution was analyzed using a flow cytometer BriCyte-E6 (Mindray, Shenzhen, China). The quantification of cell cycle distribution was carried out using the software ModFit LT™ (Verity Software House, Topsham, ME, USA). A total number of 2 × 10^4^ cells were analyzed per sample.

#### 3.2.9. Statistical Analysis

Experimental data are expressed as the mean ± standard deviation (SD). Statistical significance between values was evaluated by one-way analysis of variance (ANOVA) paired with Dunnett’s post hoc test using GraphPad Prism 5.00 software (GraphPad Software, San Diego, CA, USA) at levels of * *p* < 0.05, ** *p* < 0.01 and *** *p* < 0.001.

#### 3.2.10. Apoptosis Induction Assay

Buffy coat preparations from healthy donors were obtained from the Blood Transfusion Center in Leuven, Belgium. Peripheral blood mononuclear cells (PBMC) were isolated by density gradient centrifugation over Lymphoprep (d = 1.077 g/mL) (Nycomed, Oslo, Norway) and cultured in cell culture medium (DMEM/F12, Gibco Life Technologies, Europe, Merelbeke, Belgium) containing 8% FBS. PBMC were seeded at 28,000 cells per well in 384-well, black-walled, clear-bottomed tissue culture plates containing the test and reference compounds at five different concentrations. Propidium iodide was added at a final concentration of 1 µg/mL, and IncuCyte^®^ Annexin V Green Reagent was added as recommended by the supplier. The plates were incubated and monitored at 37 °C for 72 h in the IncuCyte^®^. Images were taken every 3 h in the brightfield and the green and red fluorescence channels, with one field imaged per well under 10× magnification. Quantification of the fluorescent signal after 72 h in both channels using the IncuCyte^®^ image analysis software allowed for the calculation of the percentage of live, dead, and apoptotic cells. All compounds were tested in two independent experiments, implying PBMC originated from two different donors.

## 4. Conclusions

Molecular hybridization is considered an interesting approach in drug design to identify new compounds that may present dual modes of action while reducing non-desired side effects. Here we have undertaken a double strategy based on the binding mode of our cyclohexanediones, exemplified by TUB015, at the colchicine site in tubulin and its almost perfect overlap with the binding mode of nocodazole, in the search for novel chemical series that inhibit tubulin polymerization and affect STAT3 signaling. First, we have identified a central core of salicylanilide that could replace the cyclohexanedione of TUB015, employing a scaffold hopping strategy, while the functionalization of both ends of this scaffold has been performed following a hybridization approach incorporating a methoxycarbonylamino, present in nocodazole and a 2-methoxyphenylamino, similar to TUB015, resulting in compound **6**. Since salicylanilides, including niclosamide, have been reported to inhibit STAT3, different anilines were also assayed to replace the 2-methoxyaniline, being particularly relevant for compound **16,** functionalized with a 3,5-diCF_3_-anilide. Compounds **6** and **16**, which showed antiproliferative activity against a panel of cancer cell lines with IC_50_ values in the low µM range, were tested as tubulin polymerization inhibitors (similarly to TUB015) and as STAT3 inhibitors, using niclosamide as a reference compound. Interestingly, compound **6**, with a 2-methoxyaniline as the amidine, inhibited tubulin polymerization with IC_50_ values close to those of nocodazole or TUB015 and arrested the cell cycle of DU145 cells at G2/M but also showed a moderate effect on inhibiting STAT3 phosphorylation. On the other hand, compound **16**, with a 3,5-diCF_3_-anilide, significantly affected STAT3 phosphorylation, inhibited STAT3 nuclear translocation, and arrested the cell cycle at G0/G1 phases, similar to niclosamide, while no effect on tubulin polymerization was observed. Thus, the substitution pattern on the aniline on the same scaffold determines the mechanism of action underlying the antiproliferative activity of this new family of salicylanilides. Interestingly, both compounds did not show significant toxicity to PBMC cells up to 100 µM, while for niclosamide there are dead and apoptotic cells already at concentrations of 1 to 3 µM. The lack of toxicity in PBMC and the involvement of two different and complementary targets in their antiproliferative activity makes these salicylanilides a promising family of anticancer compounds that will be further explored. Moreover, our results also support the general strategy that molecular hybridization may lead to new molecules being able to achieve high efficacy through a multi-target mechanism of action whilst also reducing toxicity.

## Figures and Tables

**Table 1:** Antiproliferative activity of the synthesized compounds against different tumor cell lines.

IC_50_ (µM) ^a^							
Comp	Capan-1 ^b^	HCT-116 ^b^	NCI-H460 ^b^	DND-41 ^b^	HL-60 ^b^	K-562 ^b^	Z-138 ^b^
**6**	1.3 ± 0.1	1.4 ± 3.5	2.8 ± 1.6	1.3 ± 0.5	1.5 ± 0.1	1.3 ± 0.1	1.3 ± 0.7
**12**	>100	>100	>100	>100	>100	≥84.5	>100
**13**	35.3 ± 20.1	39.8 ± 25.0	33.3 ± 0.2	≥39.4	37.3 ± 20.4	≥67.8	>100
**14**	2.5 ± 0.6	10.4 ± 1.6	15.2 ± 6.3	3.8 ± 0.2	9.1 ± 1.9	2.9 ± 1.1	13.5 ± 3.3
**15**	>100	>100	>100	≥74.1	≥89.6	≥75.8	≥88.4
**16**	6.9 ± 1.6	4.6 ± 0.07	9.2 ± 3.9	8.7 ± 1.2	4.8 ± 4.5	6.7 ± 2.4	12.2 ± 4.0
**17**	49.9 ± 28.5	≥47.1	>100	>100	3.3 ± 1.1	35.7 ± 21.0	>100
**18**	28.0 ± 0.7	71.4 ± 40.4	62.3 ± 12.0	29.9 ± 3.0	68.0 ± 1.4	26.3 ± 13.3	60.2 ± 35.9
**21**	>100	>100	>100	>100	>100	>100	>100
**TUB015**	0.06 ± 0.003	1.1 ± 0.3	0.30 ± 0.01	0.40 ± 0.007	0.6 ± 0.1	0.5 ± 0.1	0.1 ± 0.06
**Nic ^b^**	1.35 ± 0.6	0.4 ± 0.3	0.9 ± 0.3	0.45 ± 0.4	2.1 ± 0.6	0.7 ± 0.7	0.3 ± 0.4
**Noc ^b^**	0.018 ± 0.01	0.070 ± 0.04	0.20 ± 0.10	0.21 ± 0.16	0.13 ± 0.09	0.095 ± 0.04	0.088 ± 0.04

^a^ IC_50_: Concentration of compound at which 50% of cell proliferation is inhibited. Mean value of two independent experiments ±SEM. ^b^ Capan-1: pancreatic adenocarcinoma; HCT-116: colorectal carcinoma; NCI-H460: lung carcinoma; DND-41: acute lymphoblastic leukemia; HL-60: acute myeloid leukemia; K-562: chronic myeloid leukemia; Z-138: non-Hodgkin lymphoma. ^b^ Nic stands for niclosamide; Noc stands for nocodazole.

**Table 2:** Cell proliferation analysis in DU145 cells. Cell proliferation was determined using WST-1 assay after 24, 48, and 72 h incubation with serial dilutions of tested compounds **6**, **16,** and niclosamide.

Compound	Incubation Time (h)	IC_50_ (µM) ^a^
**6**	24	>30
	48	11.87 ± 0.69
	72	10.81 ± 1.83
**16**	24	13.67 ± 1.82
	48	1.95 ± 0.63
	72	1.83 ± 0.49
**Niclosamide**	24	0.94 ± 0.13
	48	0.36 ± 0.12
	72	0.25 ± 0.04

^a^ Values shown are the mean ± SD from three independent experiments, each performed in triplicate.

## Data Availability

Data is contained within the article and Supplementary Materials.

## Supplementary Materials

The following supporting information can be downloaded at: https://www.mdpi.com/article/10.3390/ph15070835/s1, Figures S1 and S2: immunofluorescence staining assay of the salicylanilide **6** against α-tubulin in HEp-2 cells and docking of compound **6** in the complex tubulin-TUB015. ^1^H and ^13^C NMR spectra of the tested compounds.
