# Supplementary material for: Hybridization Approach to Identify Salicylanilides as Inhibitors of Tubulin Polymerization and Signal Transducers and Activators of Transcription 3 (STAT3)
Source: Pharmaceuticals (Basel). 2022 Jul 6;15(7):835. doi: 10.3390/ph15070835 (PMC9318074; doi:10.3390/ph15070835)

Supplementary material related to the article

Hybridization approach to identify salicylanilides as inhibitors of tubulin polymerization and signal transducer and activator of transcription 3 (STAT3)

Marta Gargantilla [1], Leentje Persoons [2], Tereza Kauerová [3], Natalia del Río [1], Dirk Daelemans [2], Eva-María Priego [1], Peter Kollar [3]* and María-Jesús Pérez-Pérez [1]*

[1] Instituto de Quimica Medica (IQM, CSIC) c/Juan de la Cierva 3, 28006-Madrid, Spain; mgargantilla@iqm.csic.es (M.G.), natalia.delrio@iqm.csic.es (N. R.), empriego@iqm.csic.es (E.-M.P.).

[2] KU Leuven Department of Microbiology, Immunology and Transplantation, Laboratory of Virology and Chemotherapy, Rega Institute for Medical Research, KU Leuven, Herestraat 49, 3000 Leuven, Belgium; leentje.persoons@kuleuven.be (L.P.); dirk.daelemans@kuleuven.be (D.D.)

[3] Department of Pharmacology and Toxicology, Faculty of Pharmacy, Masaryk University, Palackeho tr. 1946/1, 612 42 Brno, Czech Republic; kauerovat@pharm.muni.cz (T. K.)

* Correspondence: kollarp@pharm.muni.cz (P. K.); mjperez@iqm.csic.es (M.-J.P.-P.); Tel.: +34-91-2587516.

Includes:

Computational methods S2

Figure S1: Inmmunofluorescence staining of α-tubulin in Hep-2 cells S3

Figure S2: Proposed binding pose for compound 6 at the colchicine site S4

^{1}H and ^{13}C NMR spectra of selected compounds S5

Computational methods

The 3D structure of compound **6** was generated using the tool Maestro, implemented in the Schrodinger Suite (Schrödinger Release 2017-2: Maestro, Schrödinger, LLC, New York, NY, 2017) and optimized using the tool Macromodel.

The automated docking experiment was carried out using TUB075-tubulin complex (pdb id: 6FKJ). A three-dimensional cubic grid, consisting of 50x50x50 points with a spacing of 0.375 Å, was defined at the colchicine binding site. Electrostatic, desolvation, and affinity maps for the atom types present in the ligand was calculated using AutoGrid 4.2.6 and then the Lamarckian genetic algorithm implemented in the automated docking program AutoDock4.2.20 Intra- and intermolecular energy evaluation of each configuration allowed the selection of the best-scoring solutions.

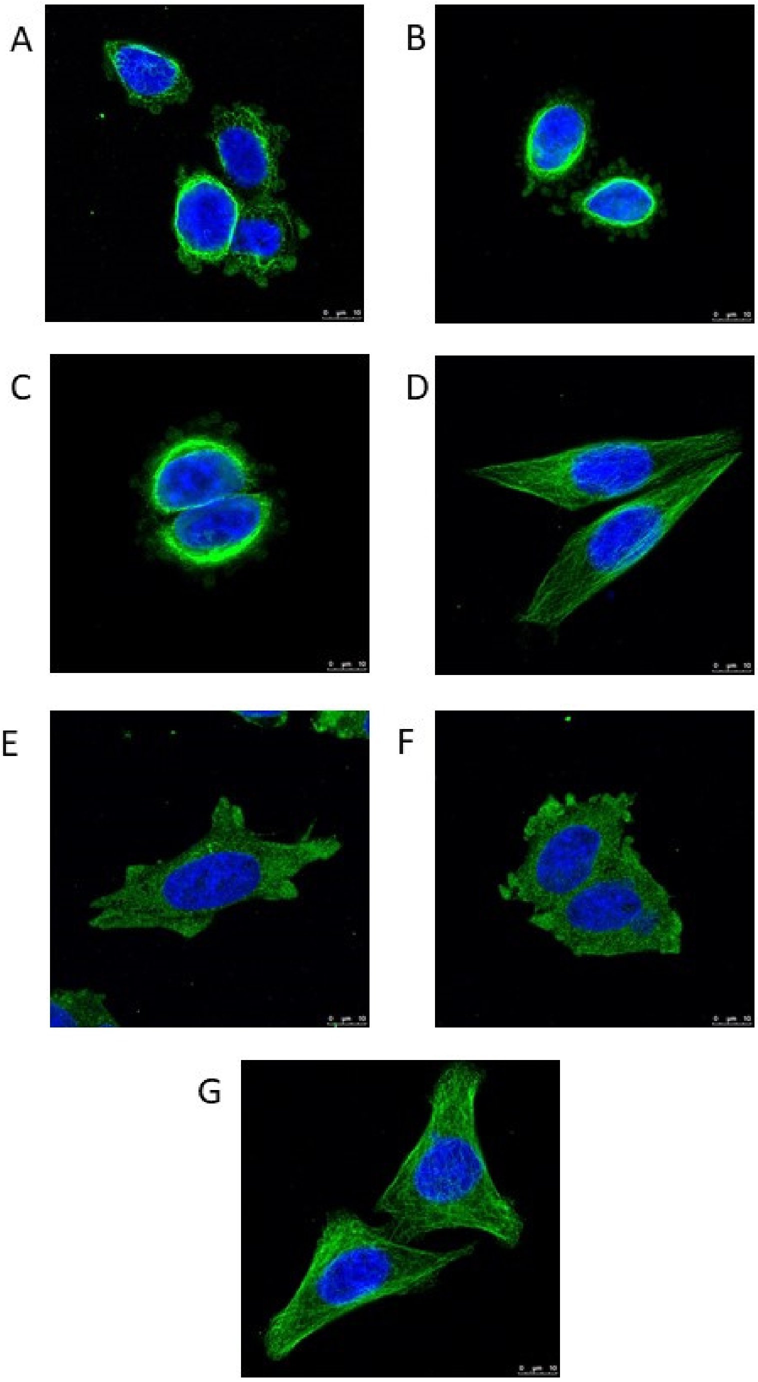

Figure S1. Inmmunofluorescence staining of α-tubulin in Hep-2 cells: (A) SAL012 at 100 μM; (B) SAL012 at 10 μM; (C) SAL012 at 1 μM; (D) SAL012 at 0,3 μM; (E) TUB015 at 10 μM; (F) nocodazole at 10 μM; (G) Control (DMSO)

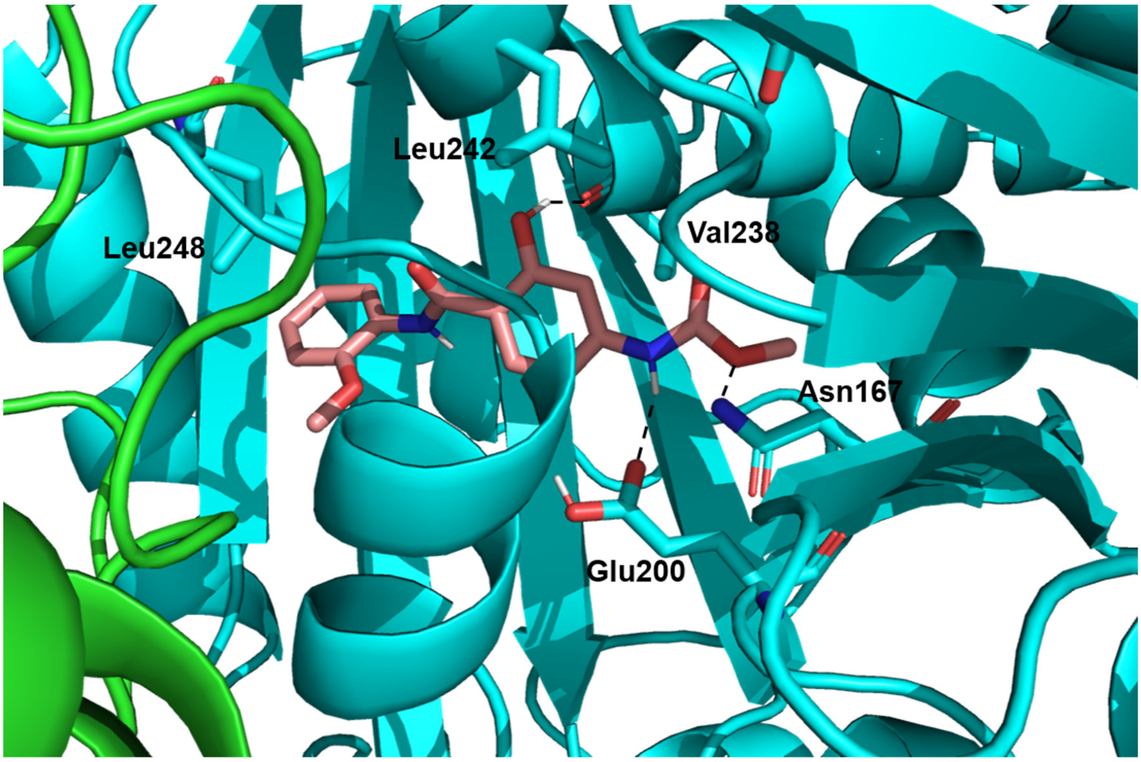

Figure S2. Proposed binding pose for compound **6** (salmon sticks) at the colchicine binding site in tubulin (PDB: 6FKJ) (α-tubulin shown in green and β-tubulin in cyan). Selected residues of the binding pocket are shown in sticks and labelled. Dashed lines represent hydrogen bonds.

Compound 6

¹H NMR (400 MHz, DMSO-d_6)

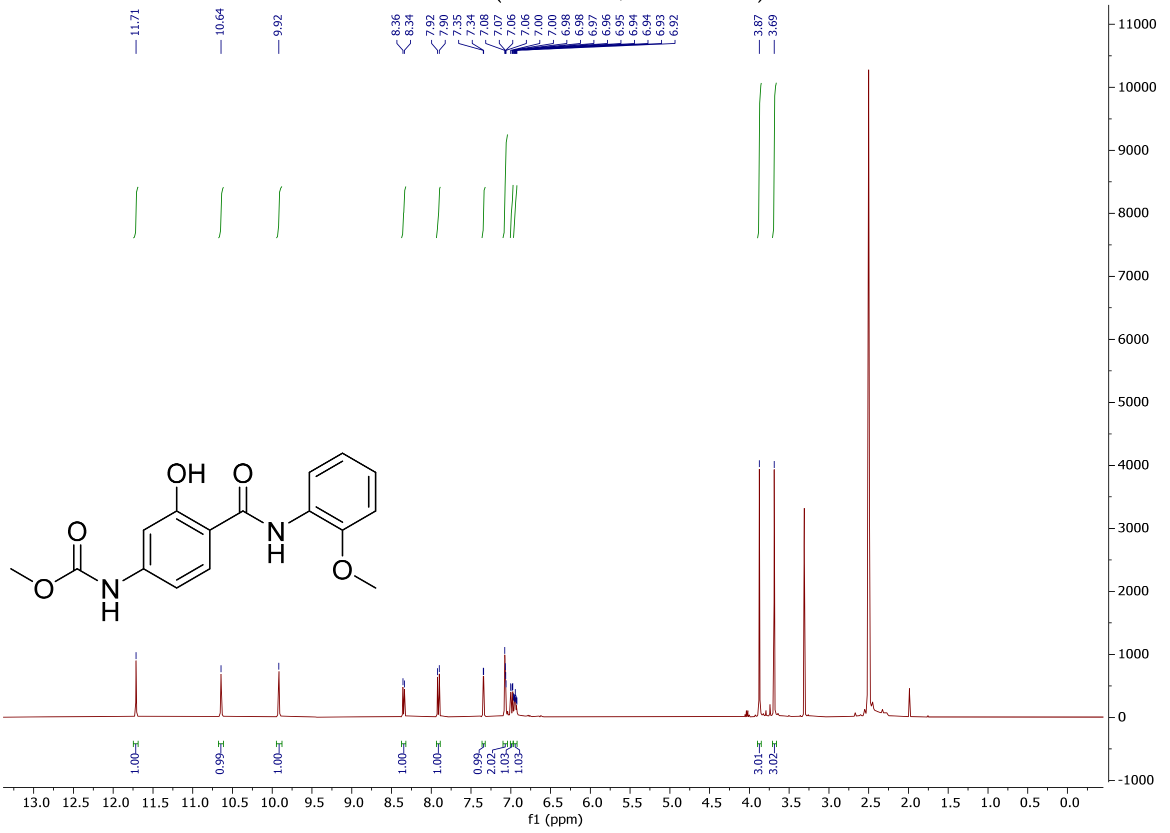

¹³C NMR (101 MHz, DMSO-d_6)

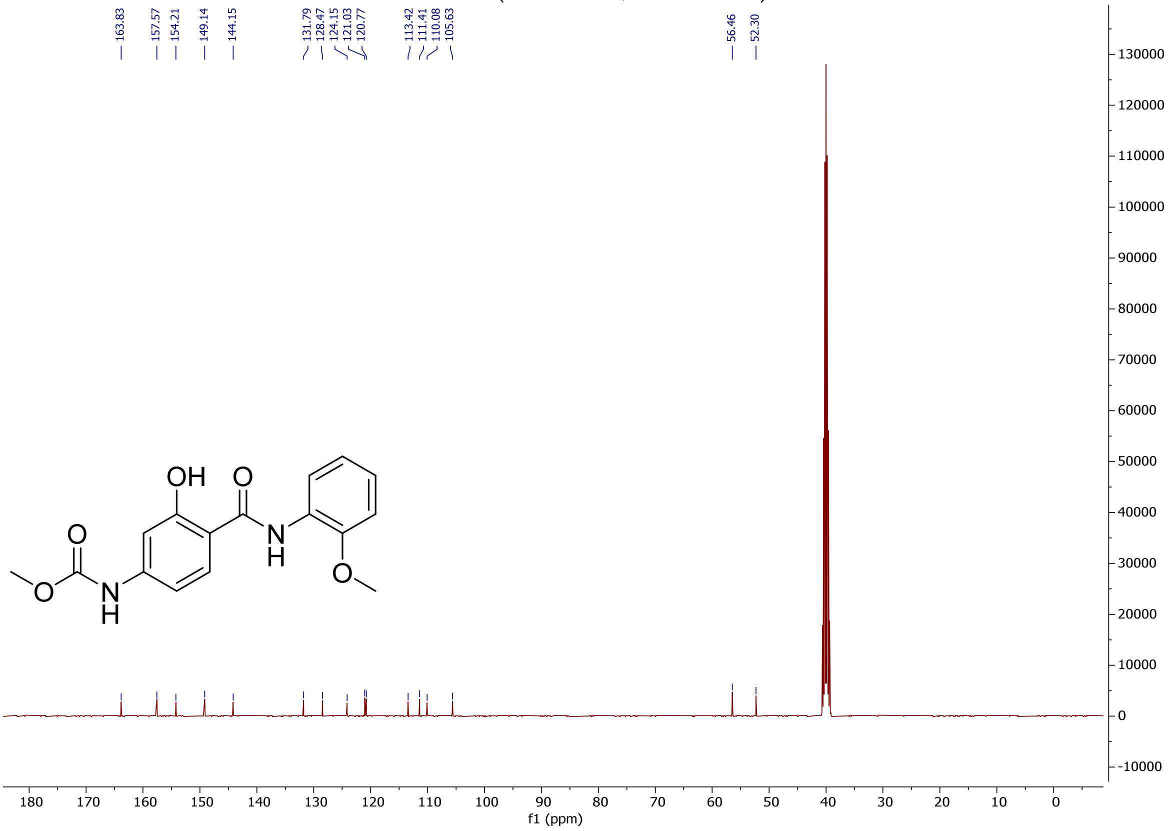

Compound 12

^{1}H NMR (400 MHz, DMSO-d_6)

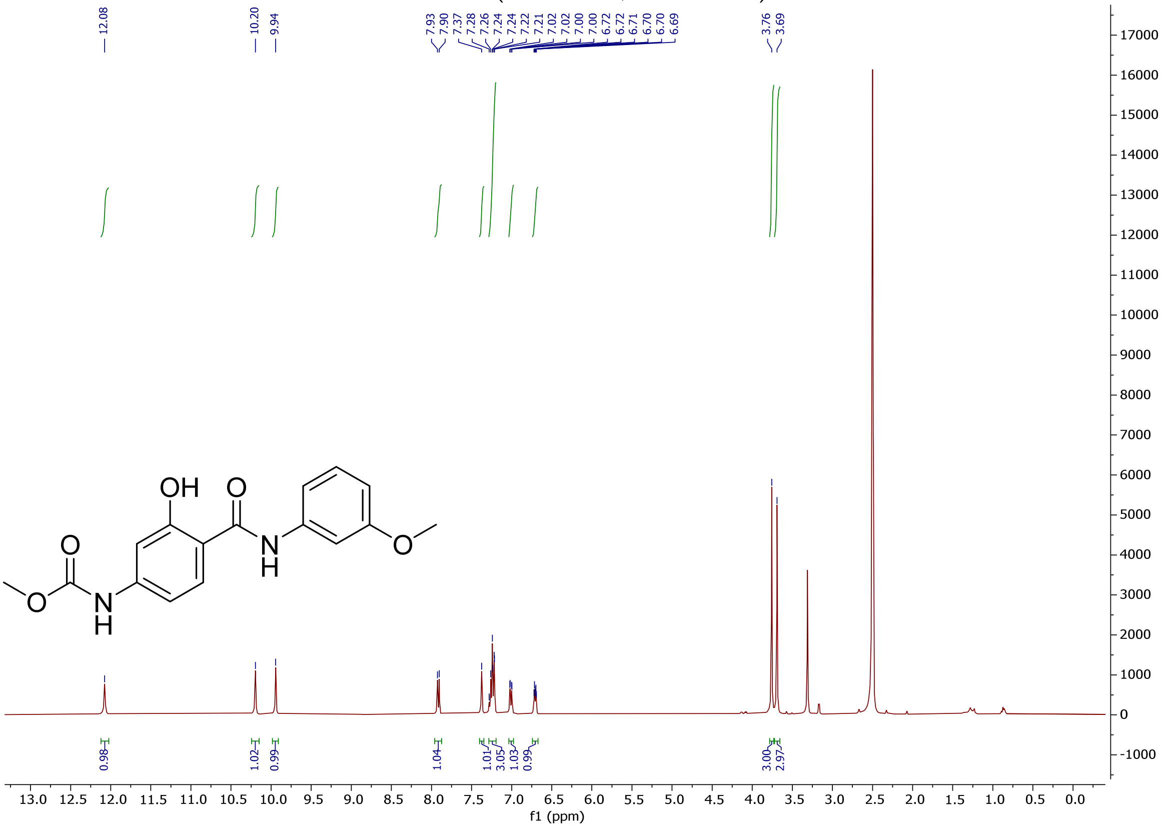

^{13}C NMR (101 MHz, DMSO-d_6)

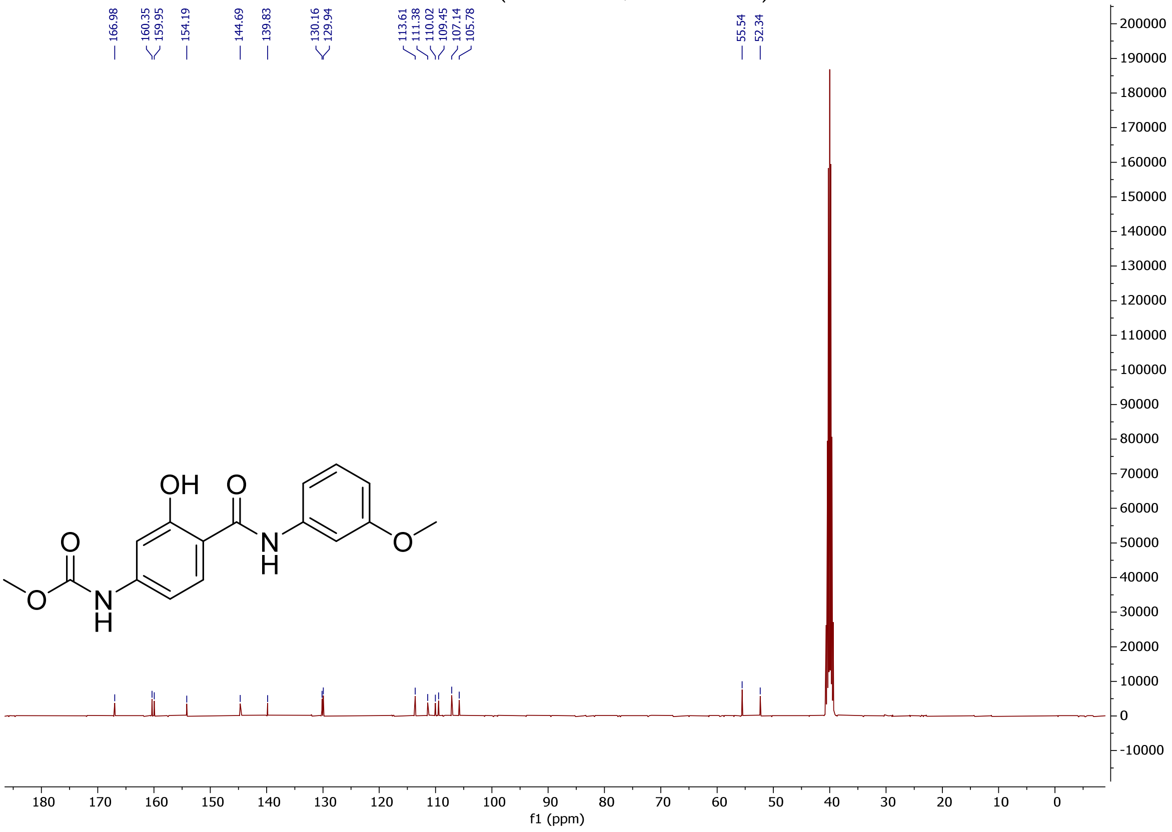

Compound 13

^{1}H NMR (400 MHz, DMSO-d_6)

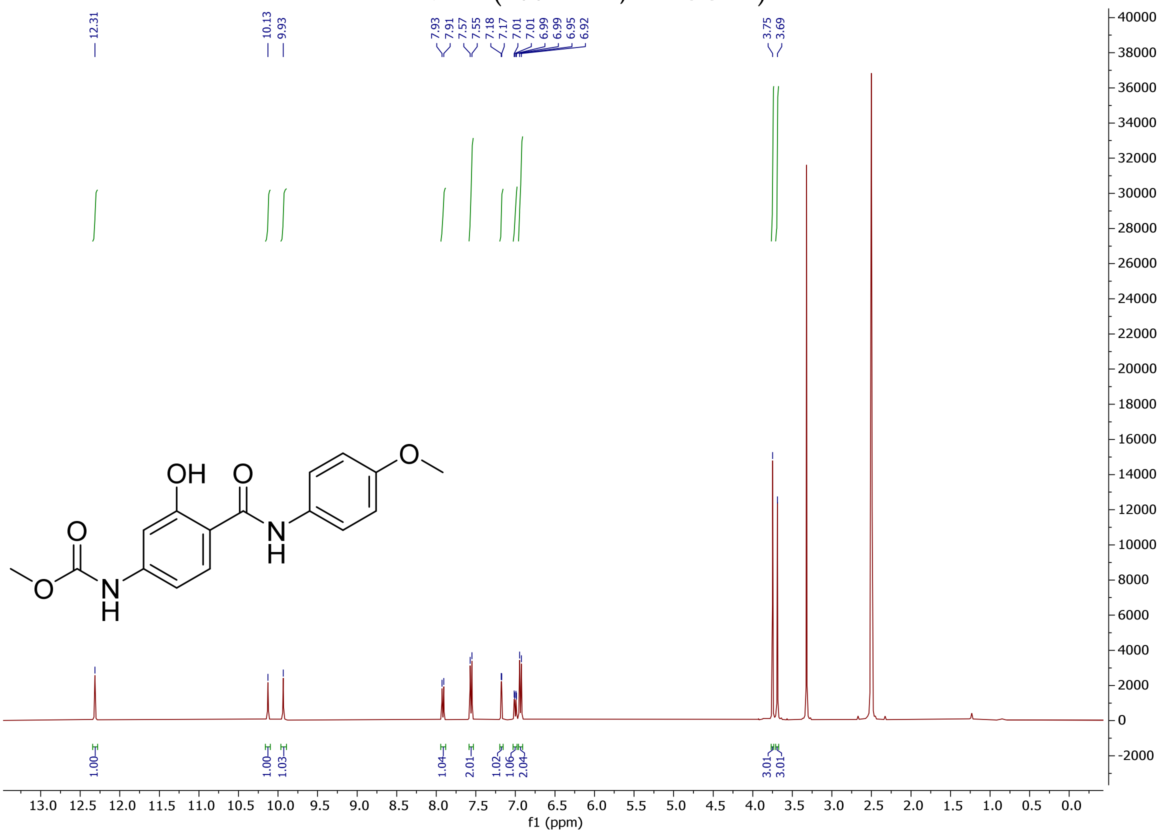

^{13}C NMR (101 MHz, DMSO-d_6)

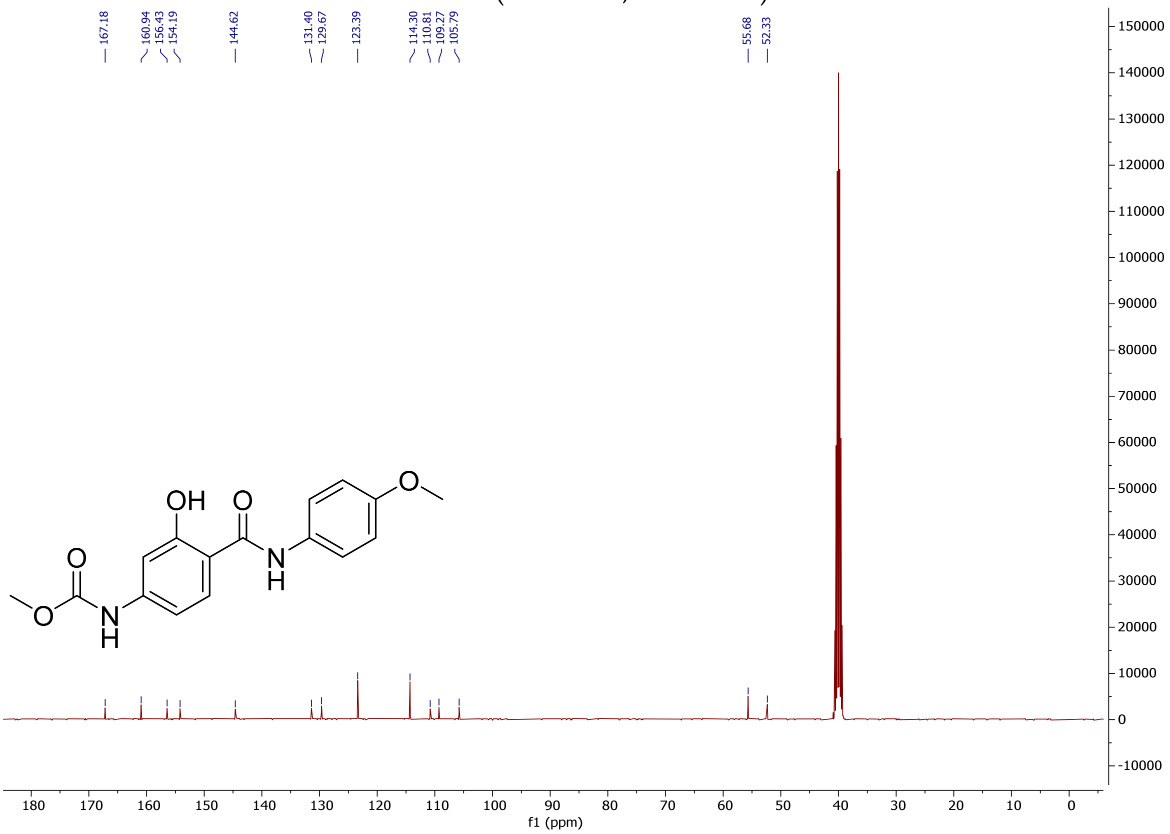

Compound 14

^{1}H NMR (400 MHz, DMSO-d_6)

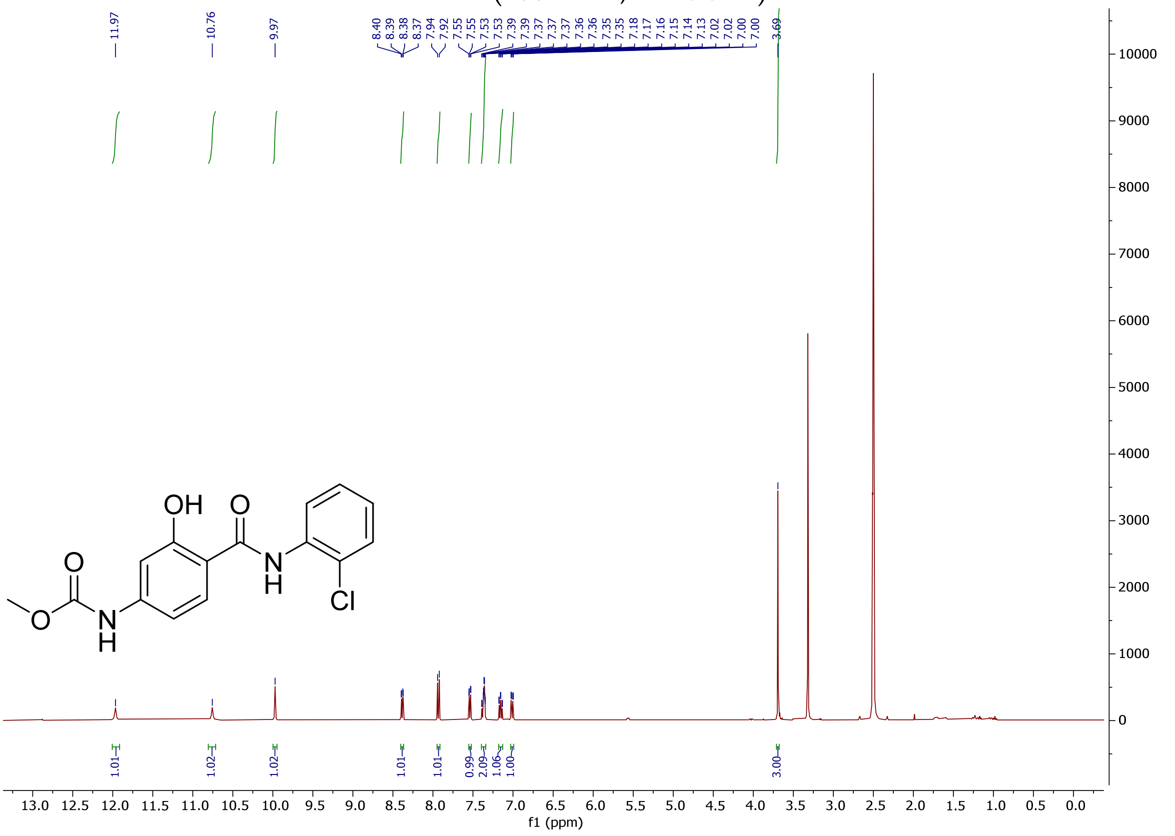

^{13}C NMR (101 MHz, DMSO-d_6)

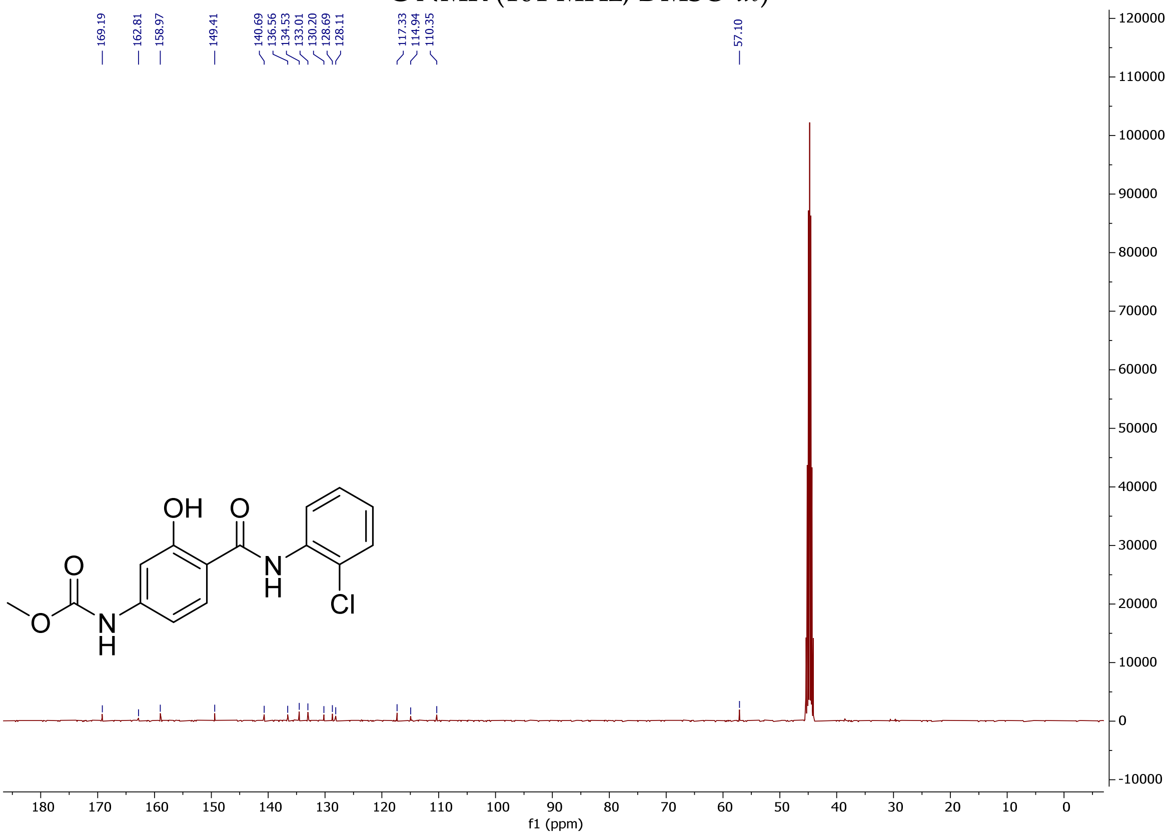

Compound 15

^{1}H NMR (400 MHz, DMSO-d_6)

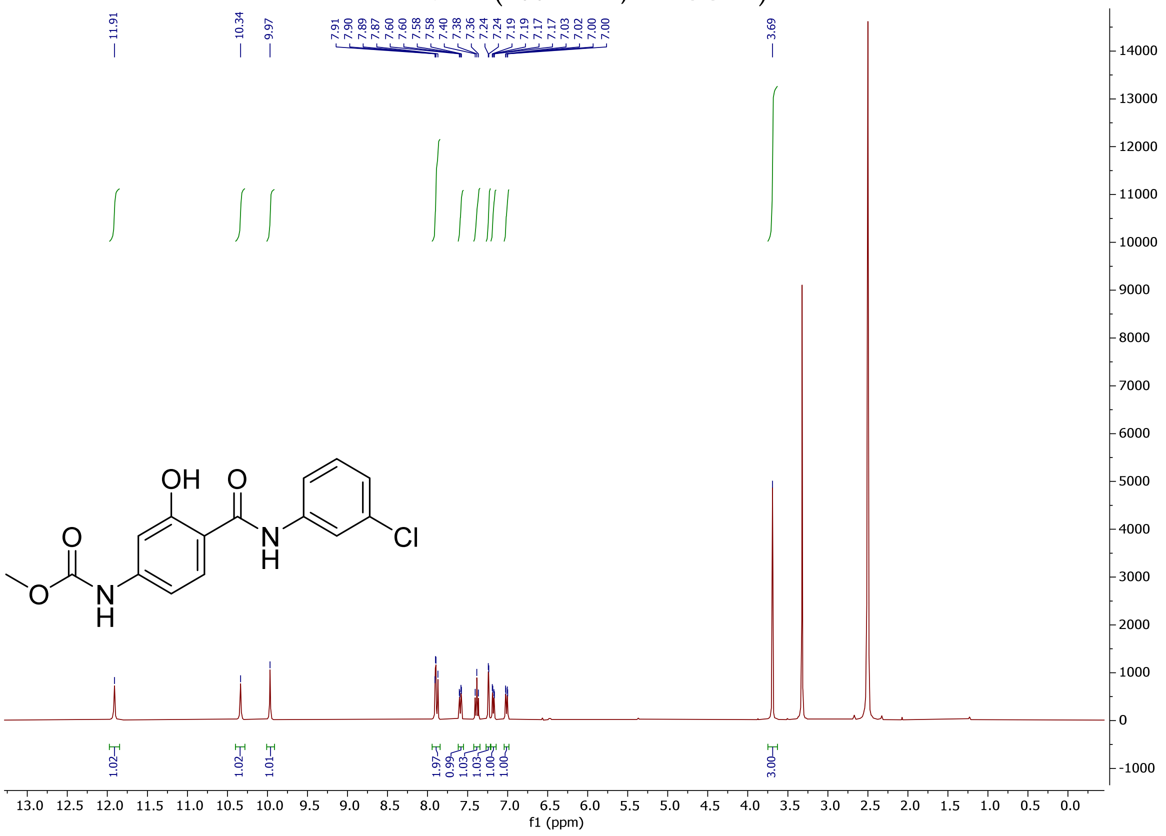

^{13}C NMR (101 MHz, DMSO-d_6)

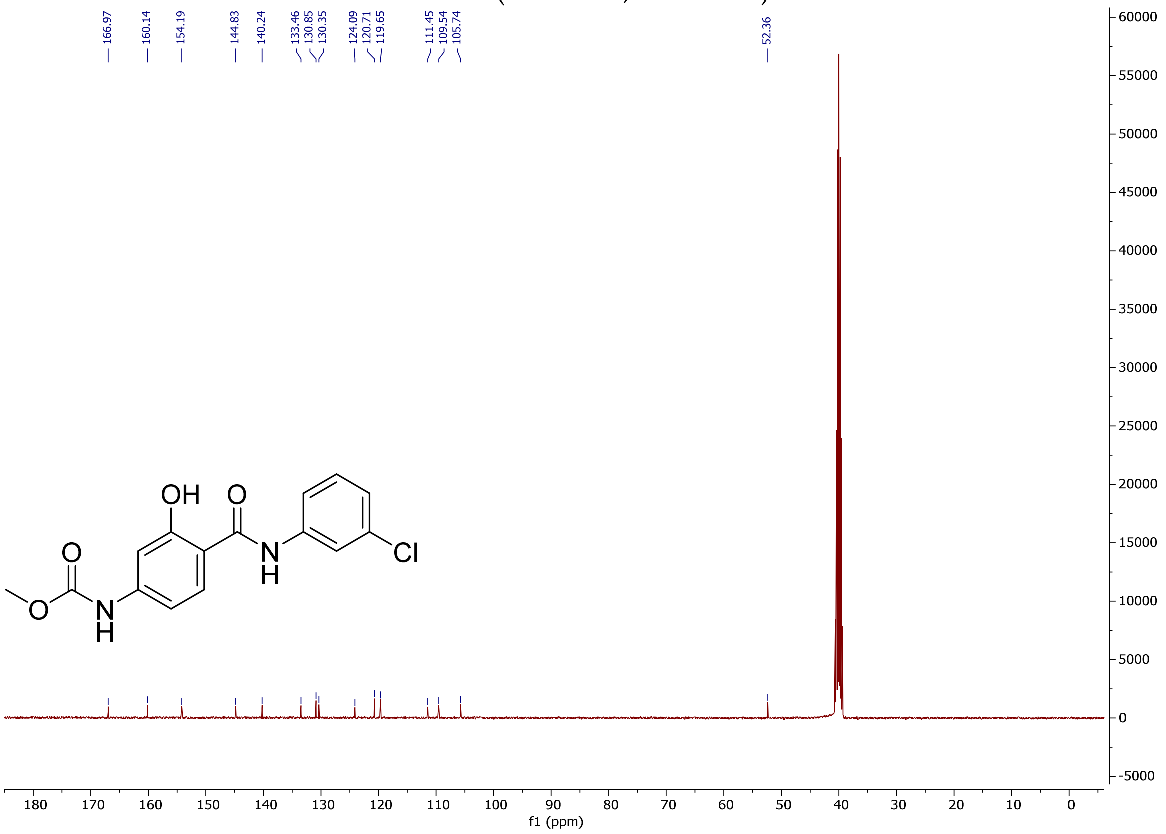

Compound 16

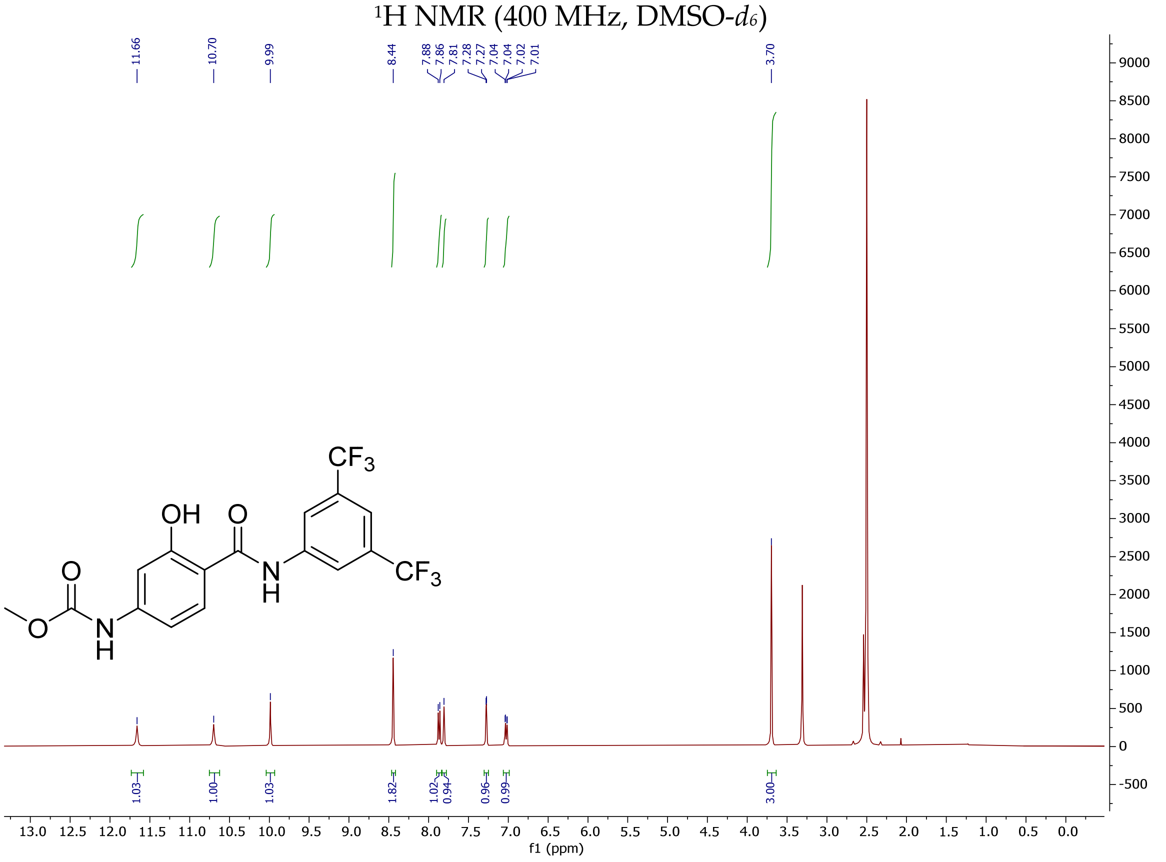

1H NMR (400 MHz, DMSO-d6)
11.66
10.70
9.99
8.44
7.88
7.86
7.81
7.28
7.27
7.04
7.04
7.02
7.01
3.70
1.03
1.00
1.03
1.82
1.02
0.94
0.96
0.99
3.00
f1 (ppm)
CF3
OH
O
O
N
H
N
H
O
CF3

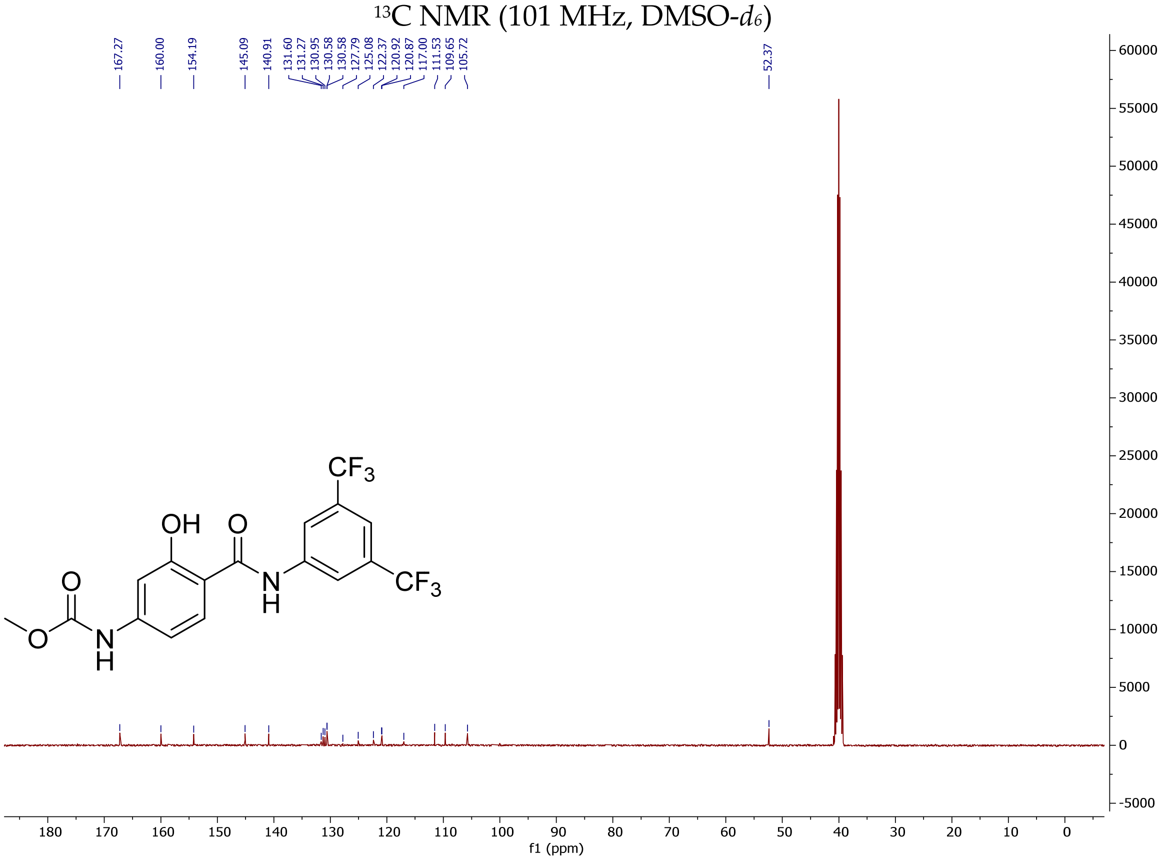

13C NMR (101 MHz, DMSO-d6)
167.27
160.00
154.19
145.09
140.91
131.60
131.27
130.95
130.58
130.58
127.79
125.08
122.37
120.92
120.87
117.00
111.53
109.65
105.72
52.37
f1 (ppm)
CF3
OH
O
O
N
H
N
H
O
CF3

Compound 17

^1H NMR (400 MHz, DMSO-d_6)

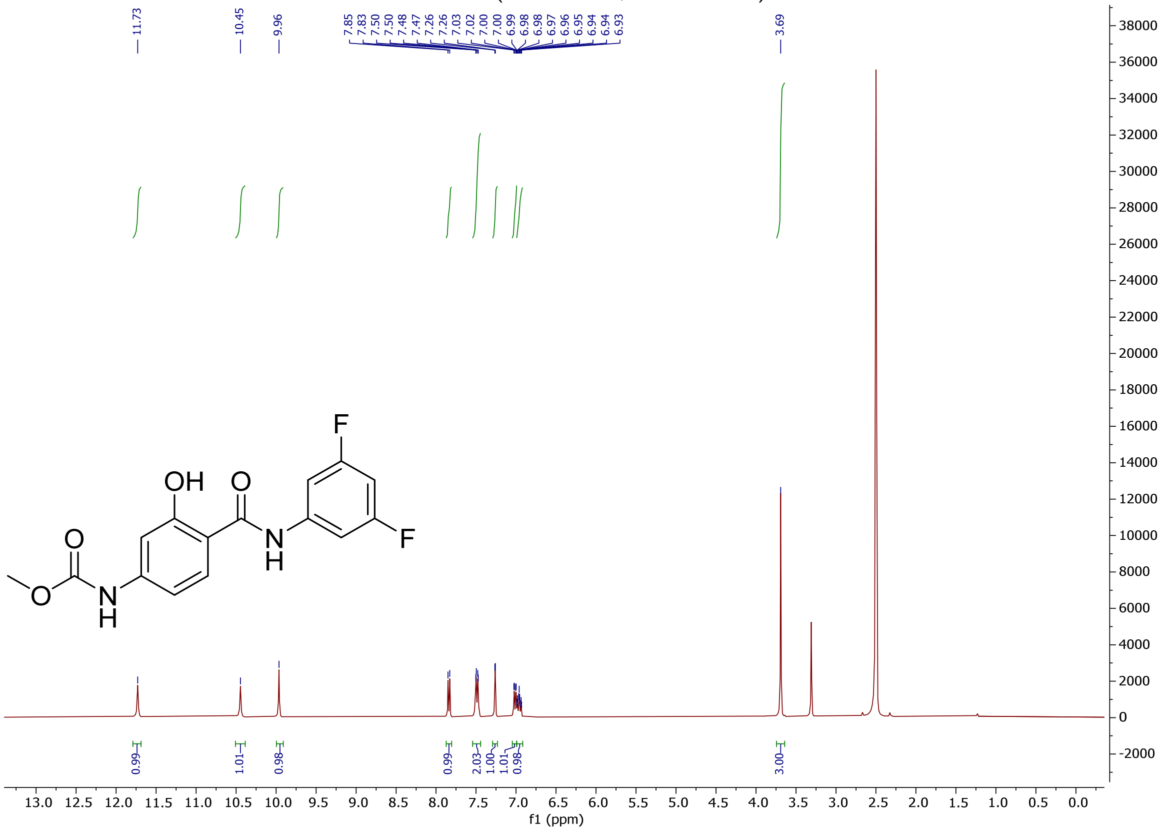

Compound 17

^1H NMR with ^{19}F decoupling (400 MHz, DMSO-d_6)

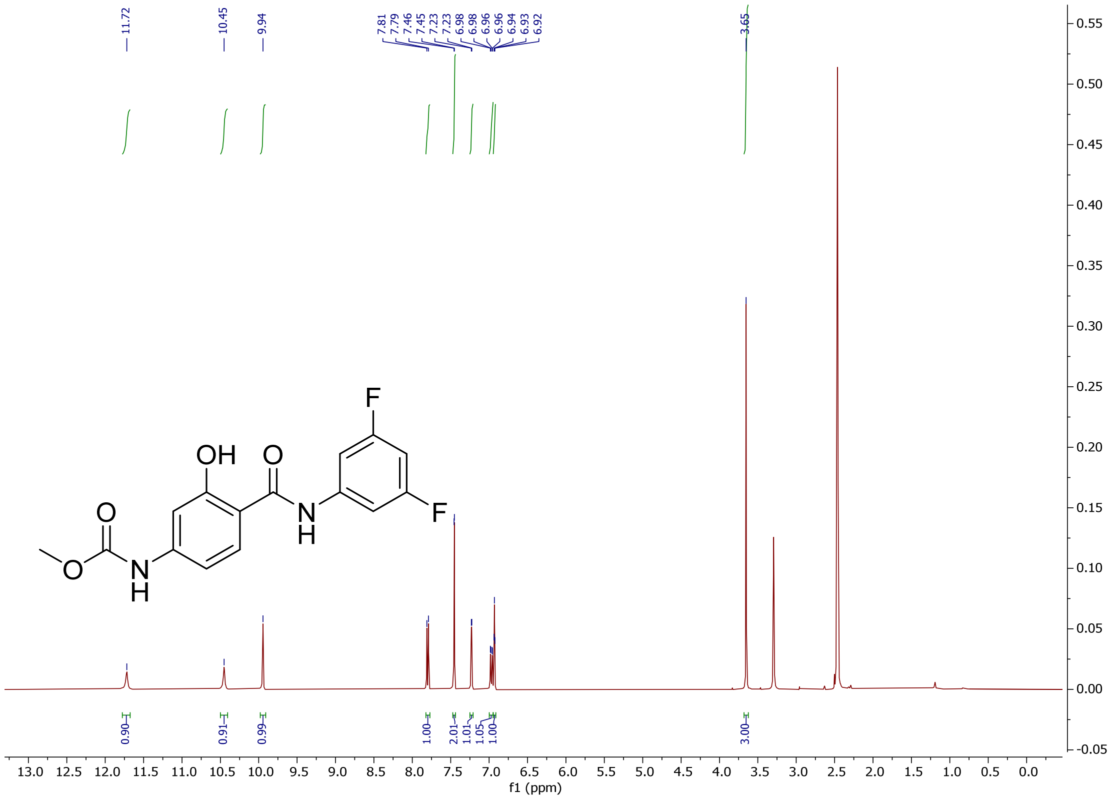

^{13}C NMR (101 MHz, DMSO-d_6)

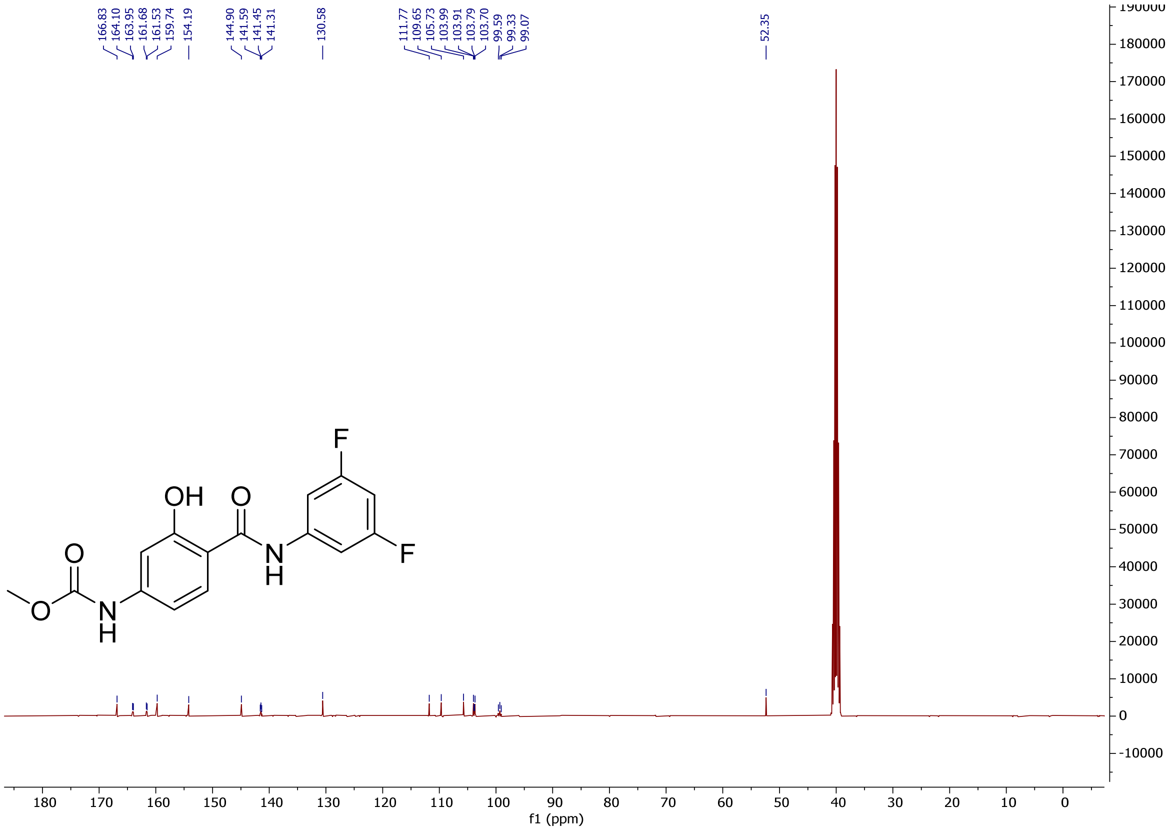

Compound 18

^{1}H NMR (400 MHz, DMSO-d_6)

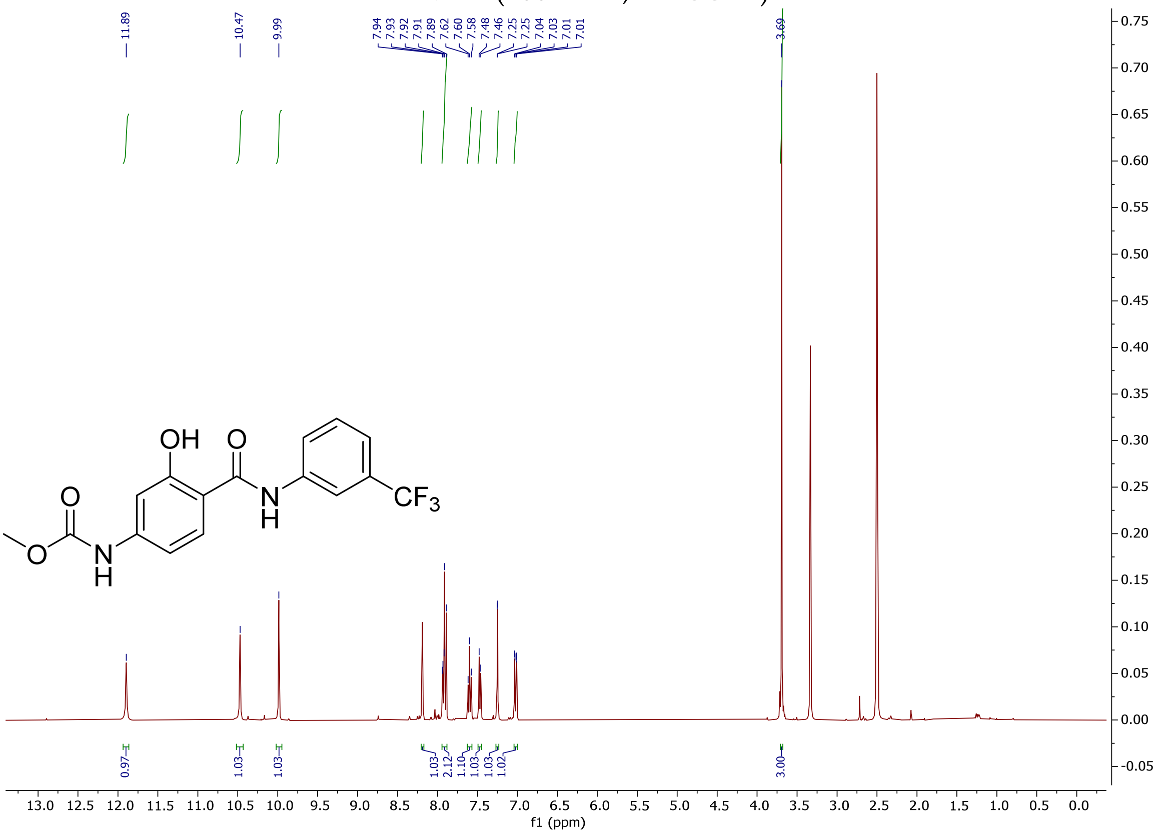

^{13}C NMR (101 MHz, DMSO-d_6)

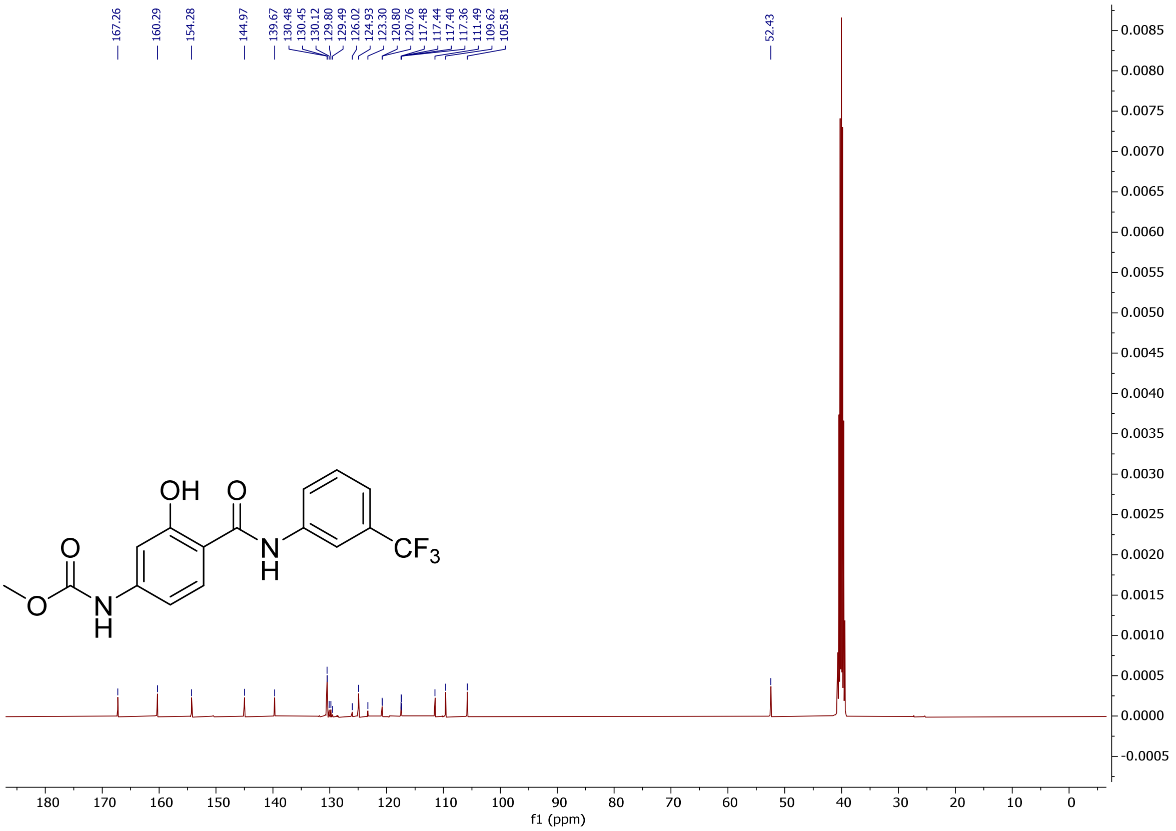

Compound 21

^{1}H NMR (400 MHz, DMSO-d_6)

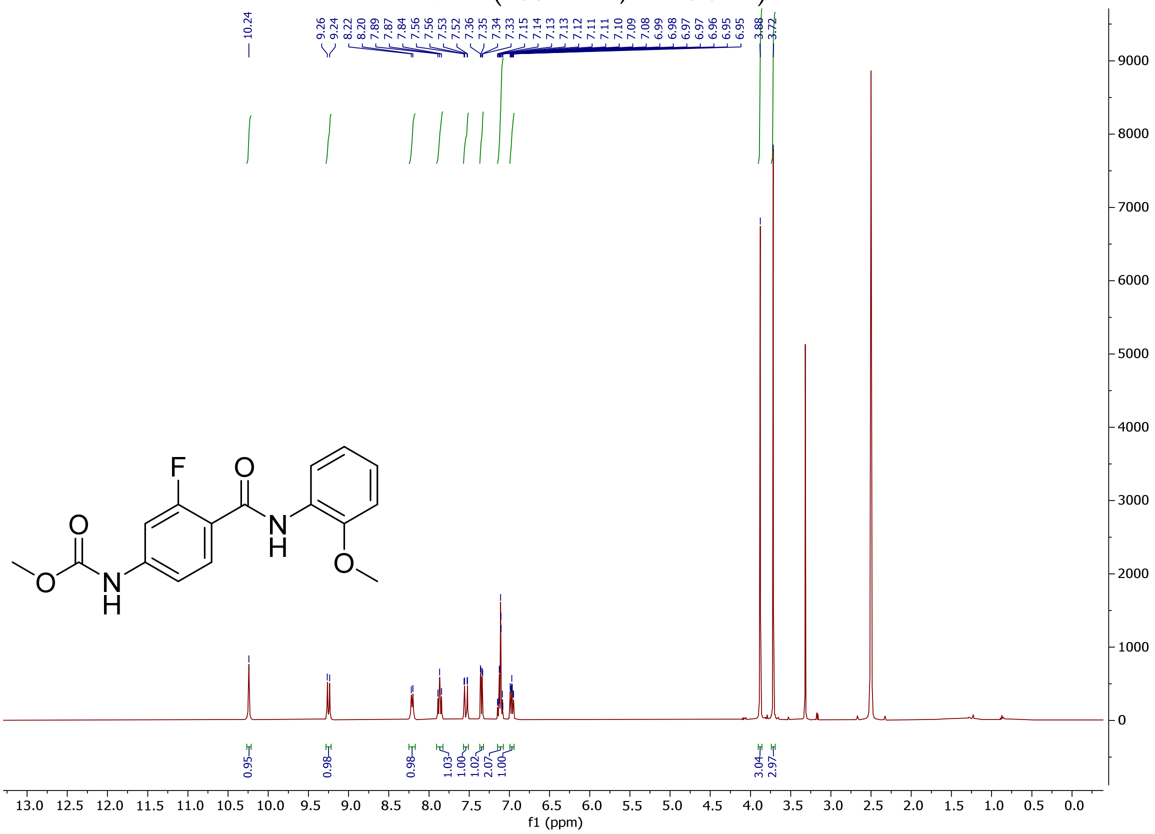

^{1}H NMR with ^{19}F decoupling (400 MHz, DMSO-d_6)

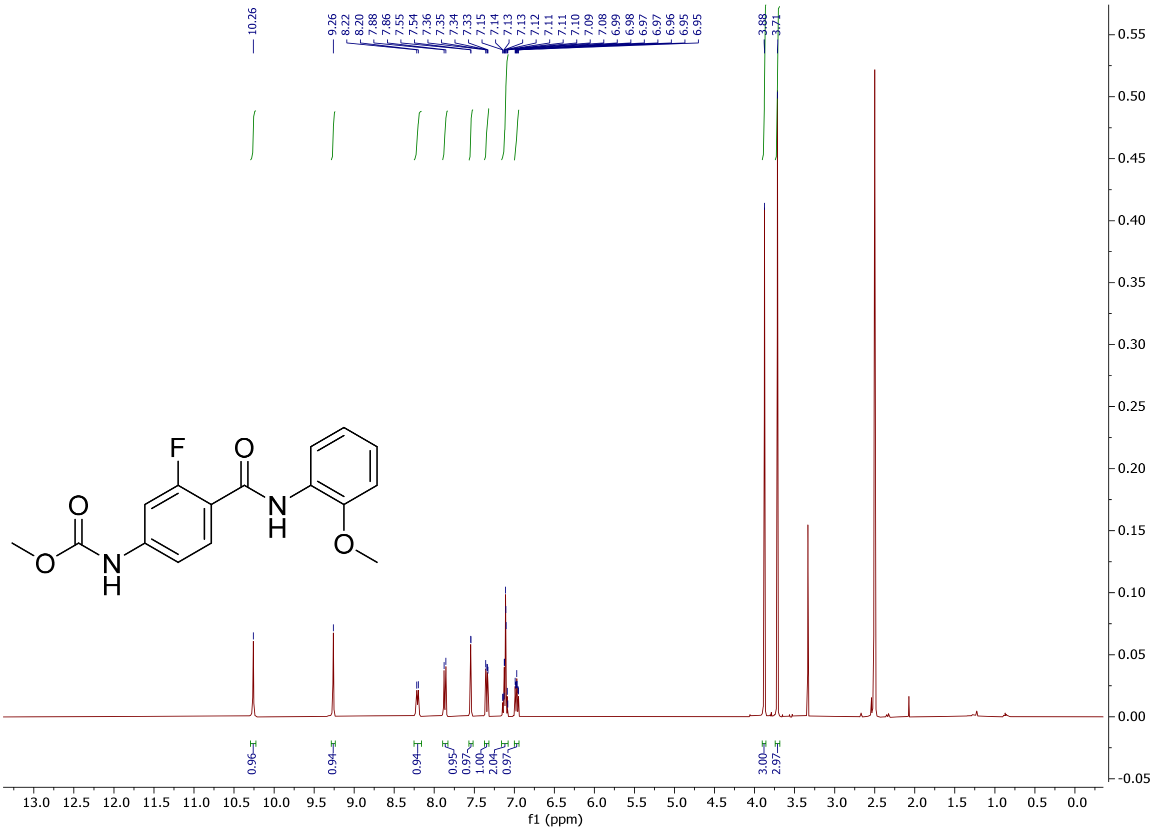

^{13}C NMR (101 MHz, DMSO-d_6)

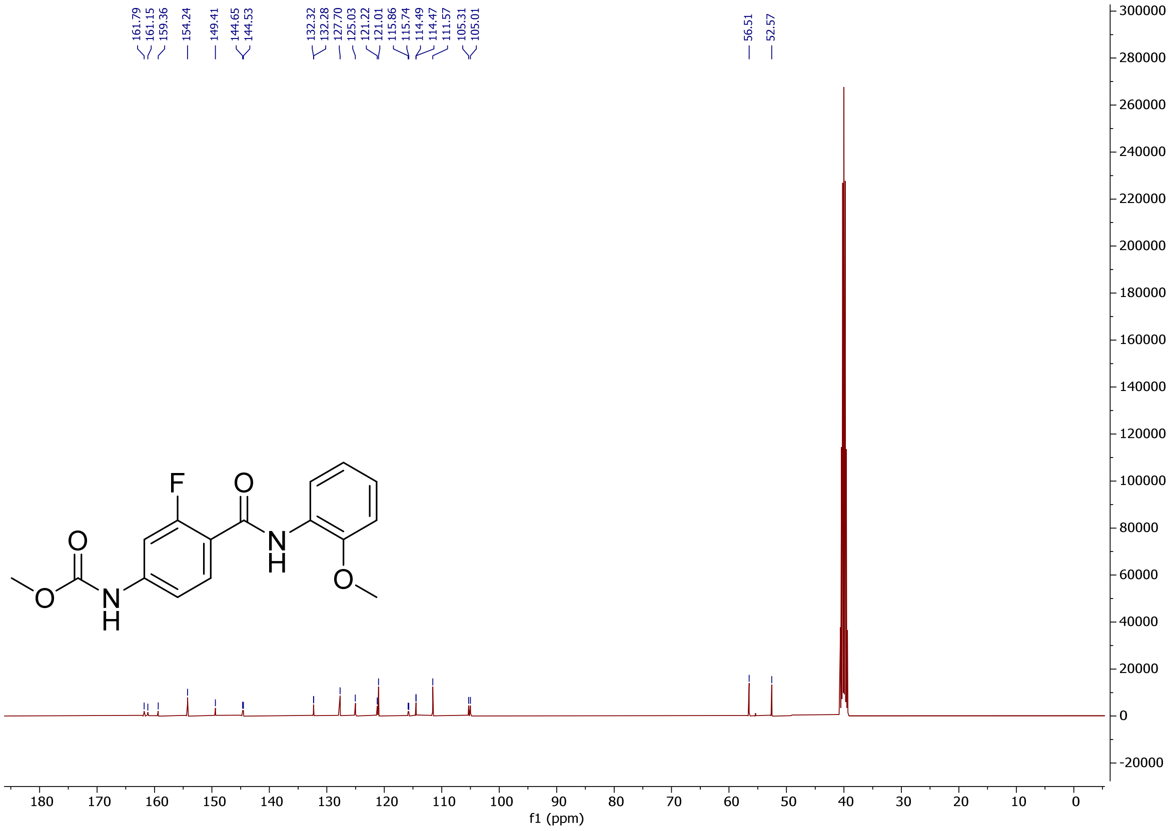

Supplement: Supplementary file 1 [file pharmaceuticals-15-00835-s001.zip › pharmaceuticals-1786146-supplementary.pdf]
